# Supplementary material for: Membrane lipid composition and rapid endocytic retrieval modulate Wingless secretion
Source: EMBO J. 2026 Aug 3;45(17):6139–75. doi: 10.1038/s44318-026-00881-2 (PMC13534635; doi:10.1038/s44318-026-00881-2)
Supplement: Supplementary file 1 [file 44318_2026_881_MOESM12_ESM.pdf]

Expanded View Figures

Figure EV1. A pipeline for WgLVs analysis.

(**A**) Manual quantification of the size of WgLVs detected by super-resolution microscopy of live GFP-Wg embryos. WgLVs are either pooled or sorted according to developmental stage ($N = 60$, 4, 19 and 16). Statistical test: one-way ANOVA with Welch correction and Dunnett's T3 multiple comparisons test. Graph bars: mean with SD. Dark diamonds indicate WgLVs observed near but not within a Wg stripe. (**B**) Anti-Wg staining of wing discs expressing endogenously tagged SNAP-Wg. The same sample was imaged with two different super-resolution setups (iSIM or SoRa) as well as standard laser confocal microscopy (SP8). The lumen of WgLVs could be resolved with iSIM and no deconvolution (dconv), indicating that WgLVs are not a deconvolution artifact. SoRa microscopy required deconvolution but was more tolerant to a range of signal intensities and sample thicknesses. (**C**) Super-resolution SoRa images of SNAP-Wg wing discs stained with anti-Wg or different SNAPtag reagents added (i) after fixation and permeabilisation or (ii) before fixation and permeabilisation. Both surface488 and surface649 reagents are technically impermeable to the cell membrane, but we found that surface48 labelled cells even prior to permeabilisation. (**D**) Quantification of the effectiveness of the GOLLUM analysis pipeline compared to gold-standard, time-consuming manual WgLV identification (see "Methods"). (**E**, **F**) Average radial profiles of anti-Wg signal of all the WgLVs (**E**) or all Wg-positive particles that did not pass the GOLLUM pipeline and hence removed from the analysis (**F**). $N = 5$, 4, 2, 5 wing discs per condition; SNAP-Wg discs with or without permeabilisation before labelling, and control (wild-type) discs treated in the same way. Error bars indicate SD. (**G**) Average radial profiles of SNAP surface649 signal on WgLVs identified using GOLLUM analysis of preparations stained with anti-Wg, $N = 5$, 4, 2, 5 wing discs per condition. (**H**) Quantification of the median SNAP surface649 intensity of all objects (WgLVs or removed particles), from the same dataset as in (**E–G**). As expected, only WgLVs from permeabilised SNAP-Wg wing discs were enriched in SNAP surface649 signal. >50 WgLVs analysed per disc. Statistical test: one-way ANOVA with Welch correction and Dunnett's T3 multiple comparisons test (SNAP-Wg permeabilised versus all other conditions). Exact P values not shown in figure: vs SNAP Perm. Rmvd. (0.003), vs SNAP No Perm. WgLVs (0.019), vs SNAP No Perm. Rmvd. (0.007), vs Control Perm. WgLVs (0.006), vs Control Perm. Rmvd. (0.005), vs Control No Perm. WgLVs (0.005), vs Control No Perm. Rmvd. (0.005). Graph bars for (**E–H**): mean with SD.

▶

 © The Author(s)

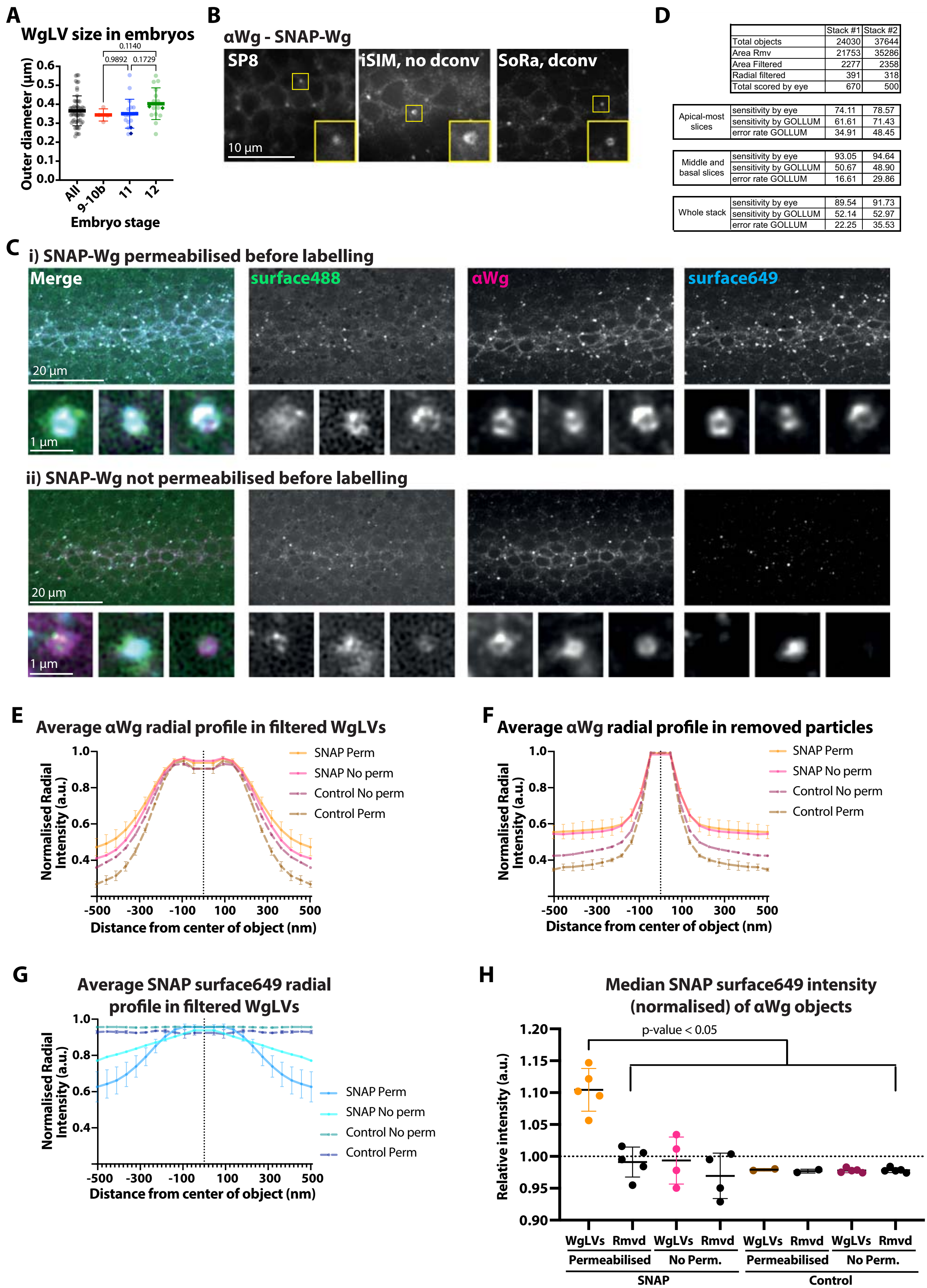

		Stack #1	Stack #2
	Total objects	24030	37644
	Area Rmv	21753	35286
	Area Filtered	2277	2358
	Radial filtered	391	318
	Total scored by eye	670	500
Apical-most slices	sensitivity by eye	74.11	78.57
	sensitivity by GOLLUM	61.61	71.43
	error rate GOLLUM	34.91	48.45
Middle and basal slices	sensitivity by eye	93.05	94.64
	sensitivity by GOLLUM	50.67	48.90
	error rate GOLLUM	16.61	29.86
Whole stack	sensitivity by eye	89.54	91.73
	sensitivity by GOLLUM	52.14	52.97
	error rate GOLLUM	22.25	35.53

© The Author(s)

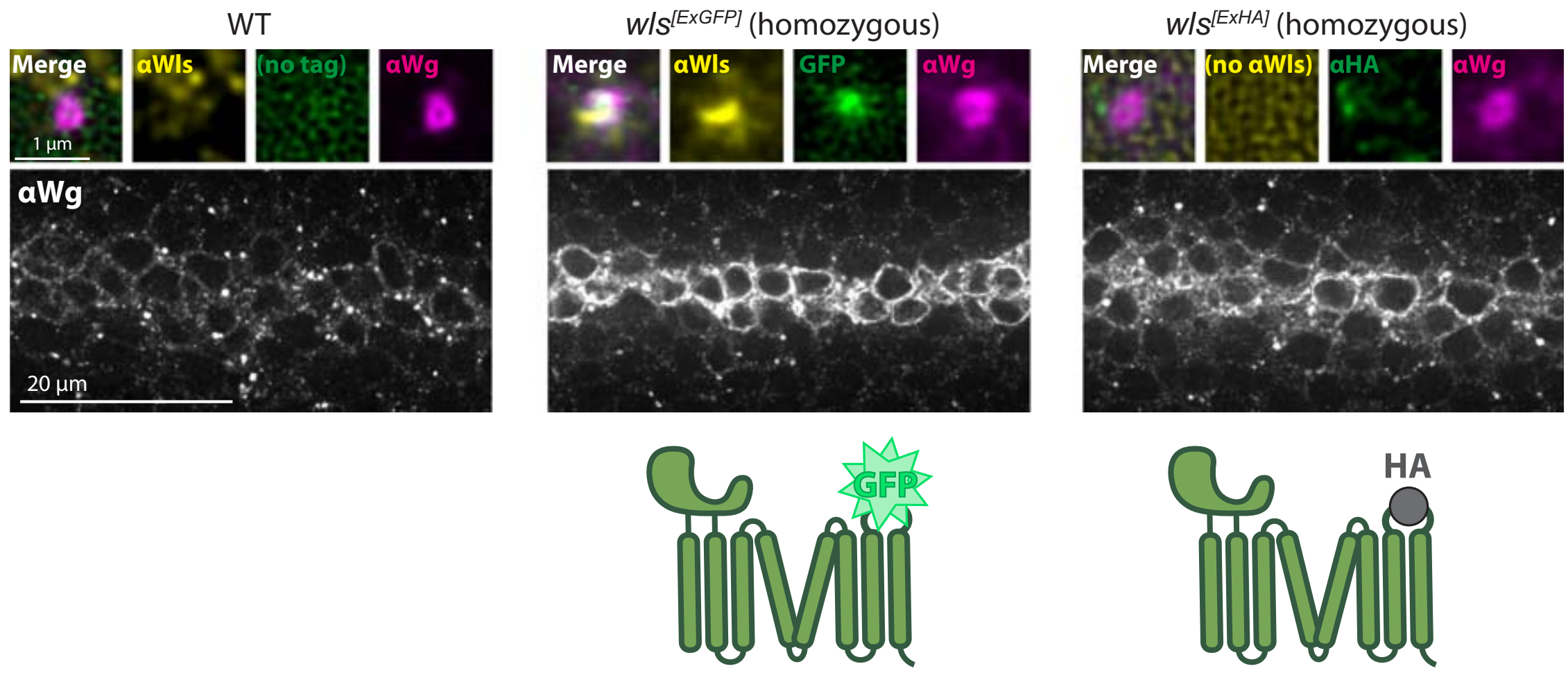

Figure EV2. Wls[ExGFP] does not readily release Wg.

Wild-type wing disc (left) compared to wing discs endogenously expressing Wls tagged with either GFP (middle) or HA (right) tags (both in the 4th extracellular loop, see diagram). For each genotype, four representative WgLV images are shown: The 3-colour merge shows anti-Wls (yellow), GFP or anti-HA (green) and anti-Wg (magenta). Note that there is no green channel signal for wild-type discs (no tag). Also, no anti-Wls signal is shown in the *wls[ExHA]* samples because anti-HA and anti-Wls are not compatible. Total Wg immunoreactivity shows abnormal Wg accumulation inside Wg-producing cells in *wls[ExGFP]* and to a lesser extent in *wls[ExHA]* wing discs.

 © The Author(s)

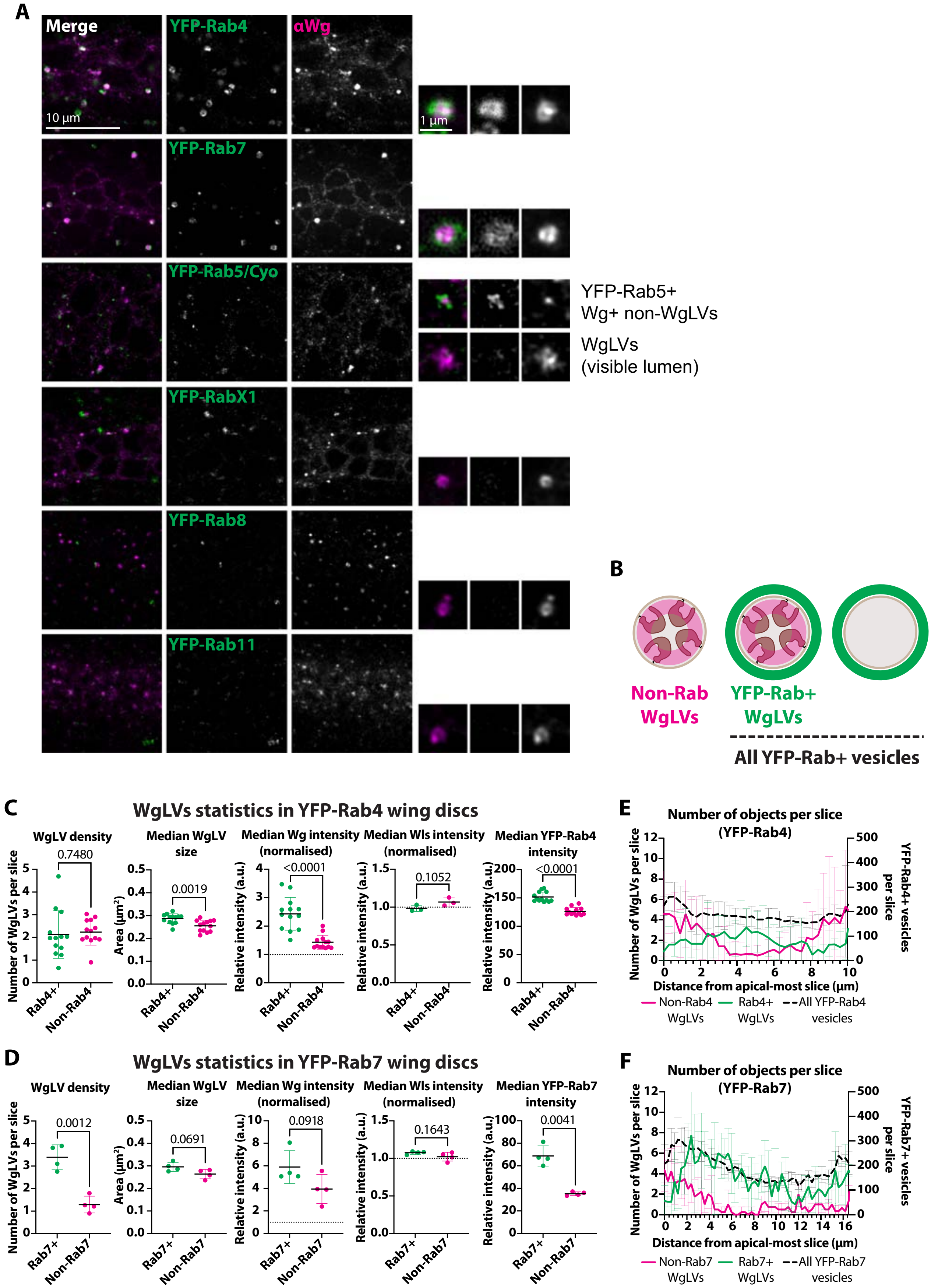
A
Merge
YFP-Rab4
αWg
10 µm
1 µm
YFP-Rab7
YFP-Rab5/Cyo
YFP-Rab5+
Wg+ non-WgLVs
WgLVs
(visible lumen)
YFP-RabX1
YFP-Rab8
YFP-Rab11
B
Non-Rab
WgLVs
YFP-Rab+
WgLVs
All YFP-Rab+ vesicles
C
WgLVs statistics in YFP-Rab4 wing discs
WgLV density
Median WgLV size
Median Wg intensity (normalised)
Median Wls intensity (normalised)
Median YFP-Rab4 intensity
Number of WgLVs per slice
Area (µm²)
Relative intensity (a.u.)
0.7480
0.0019
<0.0001
0.1052
<0.0001
Rab4+
Non-Rab4
D
WgLVs statistics in YFP-Rab7 wing discs
WgLV density
Median WgLV size
Median Wg intensity (normalised)
Median Wls intensity (normalised)
Median YFP-Rab7 intensity
0.0012
0.0691
0.0918
0.1643
0.0041
Rab7+
Non-Rab7
E
Number of objects per slice (YFP-Rab4)
Number of WgLVs per slice
YFP-Rab4+ vesicles per slice
Distance from apical-most slice (µm)
Non-Rab4 WgLVs
Rab4+ WgLVs
All YFP-Rab4 vesicles
F
Number of objects per slice (YFP-Rab7)
Number of WgLVs per slice
YFP-Rab7+ vesicles per slice
Distance from apical-most slice (µm)
Non-Rab7 WgLVs
Rab7+ WgLVs
All YFP-Rab7 vesicles

© The Author(s)

◀ **Figure EV3. Localisation of various Rabs on WgLVs.**

(**A**) A panel of wing discs expressing YFP-tagged Rabs (green) expressed from the relevant locus stained with anti-Wg (magenta) and imaged with super-resolution. All larvae are homozygous for the Rab-encoding alleles Rab except for *YFP-rab5* which is not fully homozygous viable. Only markers visibly expressed in wing discs are shown here. Note that the examples shown for Rab4 and Rab7 are the same ones as in Fig. 2A,B. (**B**) Diagram of the vesicles illustrated in (**A**). (**C–F**) Statistics of Rab4 (**C**, **E**) or Rab7 (**D**, **F**) localisation on WgLVs. WgLVs can be classified based on the presence (green) or absence (magenta) of Rab 4 or Rab7. For each disc, we calculated the median WgLV density and size, median normalised Wg and Wls intensity, and median Rab intensity throughout the image stack (**C**, **D**). We also plotted the apical-basal distribution of Rab-positive and Rab-negative WgLVs along with all Rab positive vesicles (**E**, **F**). $N = 13$ discs for YFP-Rab4 (**C**, **E**) from two independent experiments pooled together (except for anti-Wls staining, where $N = 3$ wing discs from one staining), >40 WgLVs analysed per disc. $N = 4$ discs for YFP-Rab7 (**D**, **F**), >160 WgLVs analysed per disc. Statistical test: Welch *t* test. Exact *P* values not shown in figure: YFP-Rab4 median Wg intensity (3×10^{-5}) and YFP-Rab4 intensity (3×10^{-8}). Graph bars: mean with SD.

 © The Author(s)

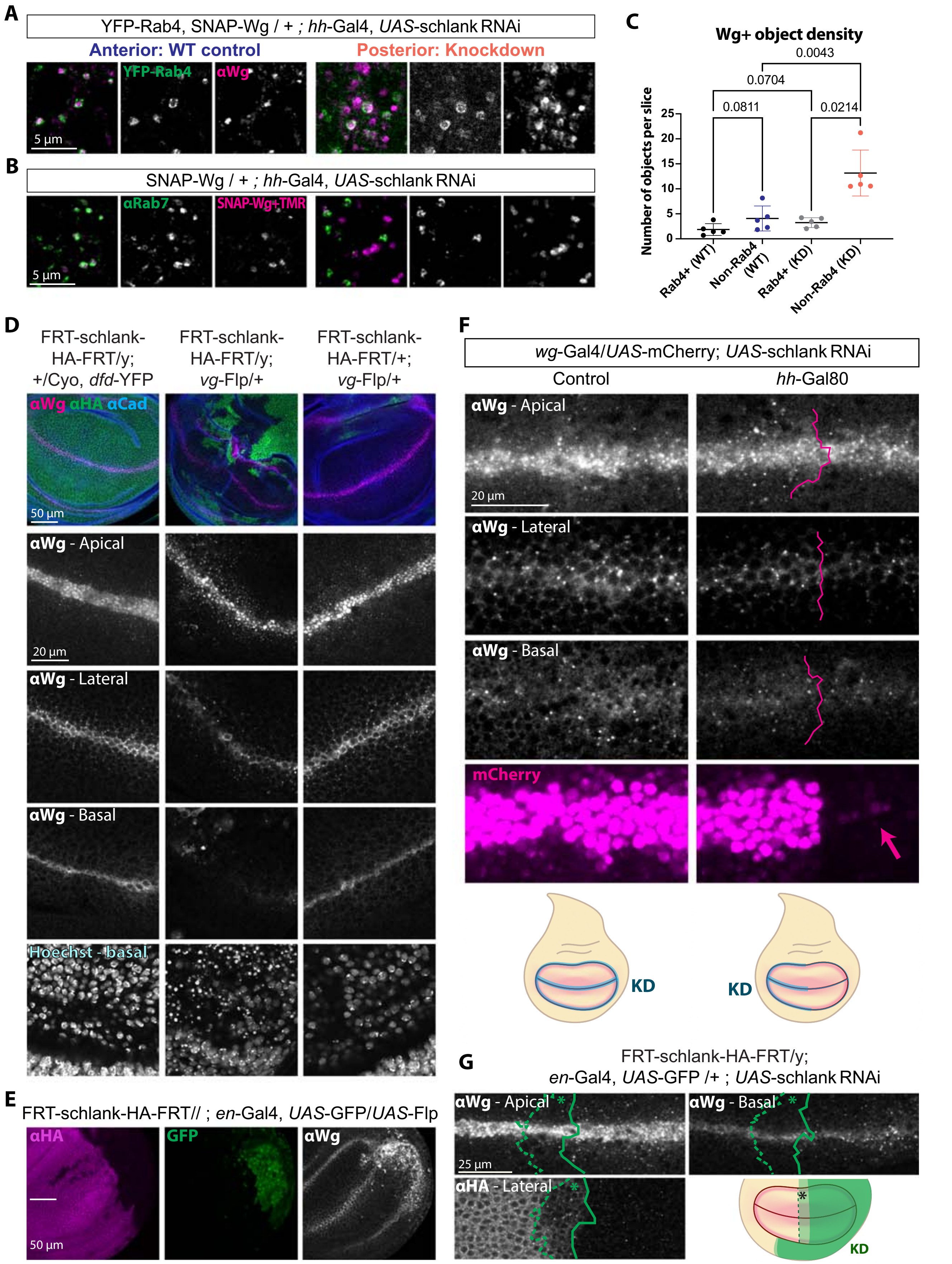
A
YFP-Rab4, SNAP-Wg / + ; hh-Gal4, UAS-schlank RNAi
Anterior: WT control
Posterior: Knockdown
YFP-Rab4
αWg
5 µm
B
SNAP-Wg / + ; hh-Gal4, UAS-schlank RNAi
αRab7
SNAP-Wg+TMR
5 µm
C
Wg+ object density
0.0043
0.0704
0.0811
0.0214
Number of objects per slice
Rab4+ (WT)
Non-Rab4 (WT)
Rab4+ (KD)
Non-Rab4 (KD)
D
FRT-schlank-HA-FRT/y; +/Cyo, dfd-YFP
FRT-schlank-HA-FRT/y; vg-Flp/+
FRT-schlank-HA-FRT/+; vg-Flp/+
αWg αHA αCad
50 µm
αWg - Apical
20 µm
αWg - Lateral
αWg - Basal
Hoechst - basal
E
FRT-schlank-HA-FRT// ; en-Gal4, UAS-GFP/UAS-Flp
αHA
GFP
αWg
50 µm
F
wg-Gal4/UAS-mCherry; UAS-schlank RNAi
Control
hh-Gal80
αWg - Apical
20 µm
αWg - Lateral
αWg - Basal
mCherry
KD
KD
G
FRT-schlank-HA-FRT/y; en-Gal4, UAS-GFP /+ ; UAS-schlank RNAi
αWg - Apical
αWg - Basal
25 µm
αHA - Lateral
KD

Figure EV4. Effects of *schlank* knockout and *schlank* knockdown in Wg-producing cells.

(**A**, **B**) Example images of SNAP-Wg and YFP-Rab4 (**A**) or anti-Rab7 (**B**) after *schlank* knockdown with *hh-Gal4*. (**C**) Quantification of the density of vesicles (WgLVs and aggregates) from either compartment in (**A**) that are YFP-Rab4 positive or negative. $N = 5$ discs, >40 vesicles analysed per disc. Statistical test: paired one-way ANOVA (comparing vesicles, positive or negative for Rab4 from the anterior and posterior compartments of the same disc) with Geisser-Greenhouse correction and Tukey's multiple comparisons test. Graph bars: mean with SD. (**D**) Wing-specific inactivation of a conditional *schlank* knockout allele (*FRT-schlank-HA-FRT*) leads to apical Wg release, loss of the basal Wg signal, and cell death. Left column: control *FRT-schlank-HA-FRT* (functional) hemizygous males. Middle column: hemizygous males with *FRT-schlank-HA-FRT* inactivated with *vg-Flp*. Right column: control females with one homolog carrying *FRT-schlank-HA-FRT* inactivated by *vg-Flp* and the other carrying wild-type *schlank*. Shown for each column are a 3-colour merge image of the wing disc stained for total Wg, Schlank-HA and cadherin. Also shown are grayscale images of total Wg immunoreactivity along the apical-basal axis, and a grayscale image of Hoechst to show pyknotic nuclei of apoptotic cells. (**E**) Inactivation of *FRT-schlank-HA-FRT* with *en-Gal4 UAS-Flp* leads loss of posterior tissue. Shown: anti-HA (magenta), GFP (reporter of Gal4 activity, green) and anti-Wg (grayscale). (**F**) Total Wg distribution after *schlank* knockdown in Wg-producing cells. Left: knockdown driven by *wg-Gal4* (as in Fig. 5B). Right: *wg-Gal4* was combined with *hh-Gal80* to inhibit Gal4 activity throughout the posterior compartment, allowing Gal4 activity in the posterior compartment only in a thin stripe where *wg-Gal4* is most expressed (as reported by mCherry, magenta, arrow). (**G**) Anti-Wg and anti-HA (Schlank) staining after *schlank* knockdown with *en-Gal4*. Here, (functional) *FRT-schlank-HA-FRT* is included to allow Schlank visualisation. As in Fig. 5C, late activation of *en-Gal4* in the domain marked by asterisk only achieved partial *schlank* knockdown.

 © The Author(s)

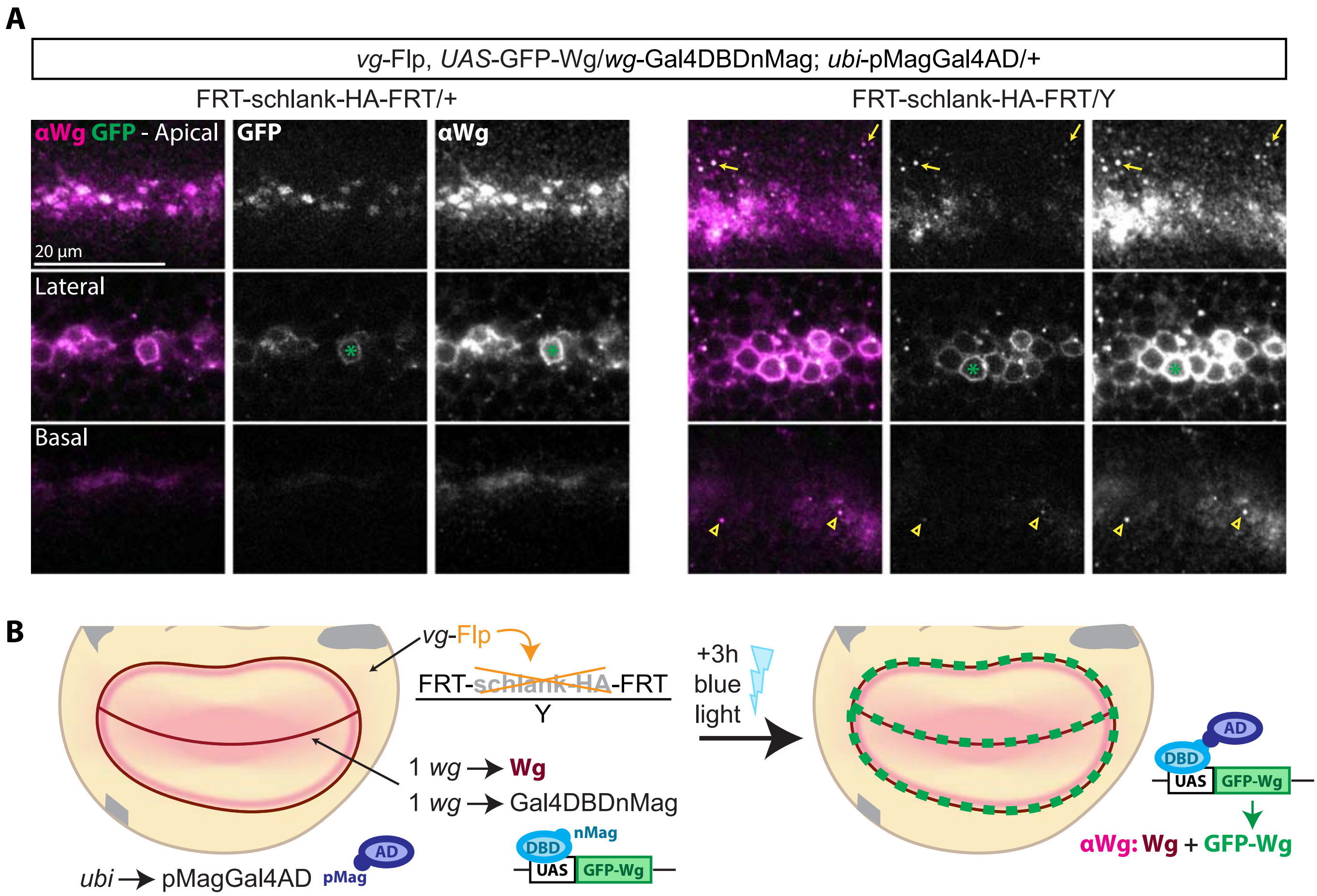

Figure EV5. Recently produced GFP-Wg accumulates in abnormal foci in *schlank* knockout wing discs.

(**A**) Total Wg and GFP-Wg signal in *FRT-schlank-HA-FRT* discs inactivated with *vg-Flp* that had GFP-Wg expression activated with Shine-Gal4 for 3 h in the normal domain of *wg* expression (see diagram). Left: control females, unaffected because one *schlank* allele is wild-type. Right: Hemizygous male disc lacking *schlank* activity. The formation of abnormal GFP-Wg punctae (yellow) both apically (arrows) and basally (arrowheads) indicates mistrafficking of recently synthesized Wg. (**B**) Diagram illustrating the genotype of the males used in this experiment. Flp (orange) was expressed directly from the *vg* promoter, leading to inactivation of *FRT-schlank-HA-FRT* and loss of HA immunoreactivity (grey) only in the pouch (see also Fig. EV4D). A few grey patches (HA-positive, not beige) are shown to illustrate that groups of wild-type cells from the hinge occasionally migrate into the pouch area. Here one half of shine-Gal4 was expressed ubiquitously while the other was expressed from the endogenous *wg* allele, ensuring optogenetic activation only in the normal domain of *wg* expression. Because of the heterogenous onset of ShineGal4 activity upon blue light exposure (Bolhaqueiro et al, under review) the level of GFP-Wg is variable (green asterisk in (**A**) and dashed green line in (**B**)). Note that anti-Wg reveals both endogenous Wg and newly produced GFP-Wg.
