# Supplementary material for: Membrane lipid composition and rapid endocytic retrieval modulate Wingless secretion
Source: EMBO J. 2026 Aug 3;45(17):6139–75. doi: 10.1038/s44318-026-00881-2 (PMC13534635; doi:10.1038/s44318-026-00881-2)
Supplement: Supplementary file 2 [file 44318_2026_881_MOESM1_ESM.pdf]

THE EMBO JOURNAL

Membrane lipid composition and rapid endocytic retrieval modulate Wingless secretion

Ines Alvarez-Rodrigo, David Willnow, Cyrille Alexandre, Claire Lebarbachon, Annabel Borg, Heather Finley, Maya Hoad Leftley, Ikram Arahouan, Yuguang Zhao, Florencia Di Pietro, Svend Kjær, Paula Booth, Reinhard Bauer, E. Yvonne Jones, Yohanns Bellaiche, and Jean-Paul Vincent

Corresponding author(s): Jean-Paul Vincent (JP.Vincent@crick.ac.uk) , Ines Alvarez-Rodrigo (ines.alvarezrodrigo@crick.ac.uk)

Review Timeline:

Transfer from Review Commons:	16th Apr 26
Editorial Decision:	29th May 26
Revision Received:	10th Jul 26
Accepted:	15th Jul 26

Review COMMONS

Editor: William Teale

Transaction Report: This manuscript was transferred to The EMBO JOURNAL following peer review at Review Commons.

Review #1

1. Evidence, reproducibility and clarity:

Evidence, reproducibility and clarity (Required)

Summary
This manuscript investigates the longstanding question of how lipidated Wnt ligands are secreted such that they can engage with their receptors. In particular it addresses how Drosophila Wingless (Wg), arguably the best studied Wnt, is secreted in the wing imaginal disc, focusing on the mechanisms of release from Wntless (Wls), a multipass transmembrane protein that chaperones lipidated Wg through the secretory system.
The initial observation is that while Wg is seen together with Wls in the secretory pathway, within Wg secreting cells there is also a population of large vesicles that are 'lined with Wg (WgLVs) that do not contain Wls. Interestingly, these vesicles appear to become coated with both Rab4 and Rab7 as they move from apical to basal within secreting cells. However, blocking endocytosis inhibits formation of WgLVs and instead leads to abnormal Wg release apically, suggesting that WgLV formation requires secretion and re-endocytosis of Wg, and in the absence of endocytosis Wg is ectopically released. The authors further show that abnormal apical Wg release and reduced Wg signaling occurs upon knockdown of Schlank (which reduces ceramide synthesis), hence implicating membrane lipid composition as an important factor in Wg trafficking. The abnormal release of Wg in Schlank knockdown is further increased by loss of the Wg-binding glypican Dlp, and decreased upon Dlp overexpression, supporting the idea of the abnormal Wg being present in insoluble aggregates.
Major points
None
Minor points
• I'm unsure whether it is a deliberate stylistic choice to use the feminine (punctae) rather than the masculine plural (puncta) of punctum throughout the manuscript, but some might say that it's about time
• p.7 "We conclude that reduction of ceramide synthesis interferes with Wg targeting to WgLVs as well as signalling" I think the authors say this, because of the appearance of abnormal apical Wg particles away from the expression domain, suggesting abnormal apical release of Wg. What puzzles me is that in the "lateral" sections I see lots of Wg signal in the knockdown region, in fact I think more than in the control region. At face value this implies more Wg in WgLVs, but the image is very small so it's not easy to interpret. Could the authors clarify whether there is less Wg in WgLVs upon schlank knockdown and why the Wg signal is so strong in all the sections in the knockdown region?

• p.9 "Super-resolution microscopy of discs endogenously expressing SNAP-Wg labelled with SNAP-Cell TMR-Star shows that Dlp localizes to some, but not all, WgLVs (Fig6A)" This is quite frustrating to interpret as a reader, as the only large hollow particles shown do seem to have some Dlp associated with them. I'm sympathetic to the idea that the authors should be able to make qualitative judgements regarding colocalization of proteins (i.e. that not every statement in a manuscript requires detailed quantitation and statistics), but the best examples shown here do not seem to support the conclusion?
• p.9 Fig.6C - I think I've also missed why in the dlp[KO] experiment, the wt control region (green) has a much higher WgLV density than the three wt controls (gray). If "WgLVs form in the absence of Dlp (dlp[KO]) ... within the wild-type range", shouldn't all conditions look the same?
• p.10 Fig.7D - quite hard to see much here, I wonder if there's a way to show this that doesn't look like almost wholly black panels with very rare white pixels that are hard to see?

2. Significance:

Significance (Required)

General assessment

This is technically leading-edge work that pushes light microscopy and precise genetic manipulation to their limits. The authors put a great deal of effort into quantifying their data and carrying out statistical analyses (which as far as I can assess, all look appropriate). In addition, to test their hypotheses they also introduce new tools, most notably an optogenetic system to inactivate clathrin, which forms a nice complement to the commonly used shibire[ts] temperature sensitive Dynamin mutation commonly used to block endocytosis.
Mechanisms of Wnt function is a complicated and controversial area of research, and this study makes important contributions to our understanding. Particular strengths are that the work is done in an in vivo physiological context, the quality of the imaging and quantitation and the sophisticated genetic manipulations and matching controls that are used.

Advance

The authors are able to draw a number of well-supported conclusions, specifically that Wg is normally secreted apically together with Wls, then re-endocytosed in Rab4/Rab7 coated vesicles that are apparently required for Wg basal release. Moreover, the re-endocytosis partially fails when ceramide synthesis is blocked (suggesting an important role for

membrane composition), and the failure of re-endocytosis results in the abnormal apical release of particles that most likely contain aggregates of insoluble Wg.

Audience

This will obviously be of interest to the large field of people who work on Wnt signaling, but also to people generally interested in mechanisms of ligand secretion and intracellular trafficking.
I have reviewed this work from my perspective as a Drosophila development biologist with an interest in cell signaling but no specific expertise in Wnt secretion. I have not attempted to review the details of the WgLV image processing pipeline.

3. How much time do you estimate the authors will need to complete the suggested revisions:

Estimated time to Complete Revisions (Required)

(Decision Recommendation)

Less than 1 month

4. *Review Commons* values the work of reviewers and encourages them to get credit for their work. Select 'Yes' below to register your reviewing activity at [Web of Science Reviewer Recognition Service] (formerly Publons); note that the content of your review will not be visible on Web of Science.

Web of Science Reviewer Recognition

Yes

Review #2

1. Evidence, reproducibility and clarity:

Evidence, reproducibility and clarity (Required)

Summary

This paper explores the movement of Wg and its intracellular chaperone, WLS, in the Drosophila wing disc. Using novel tools, the authors describe a pathway in which Wg is

shuttled to the apical membrane, re-endocytosed, and dissociated from WLS. This forms Rab4/7 positive vesicles in which Wg accumulates. From here, WLS may be recycled, and Wg presented on the basal membrane, potentially by glypicans such as Dlp. Interference of endocytosis therefore limits the formation of these large Wg vesicles at the apical surface and stunts the dissemination of Wg to receiving cells from the basal membrane, limiting signal activation. The development of Clathrin optogenetic tools is a clear highlight of the work and has great potential for use in future study. However, there are some aspects that require re-visiting before publication, particularly concerning the clarity and impact of the work.

Major comments

1. Aggregation vs vesicle identity remains unclear

The interpretation of the super-resolution microscopy analysis of those "doughnut-shaped structures or puncta needs further deeper analysis as these structures could represent:
- stalled carriers or EVs,
- aberrant endosomal subdomains,
- membrane blebs with altered lipid composition.

Additionally, the authors used FTY72 ceramide synthase inhibitor in L-cells and treated Wnt-reporter cells with conditioned medium from L-cells. The authors should demonstrate in this system that the levels of Wnt3a in the conditioned media are not altered following FTY72 treatment. Could they also provide some imaging data suggesting that the Wnt3a produced by the cells is being released as aggregates?
2. Overinterpretation of "Wg Alone" in WgLVs

The authors conclude that Wg is present in Wg-lined vesicles (WgLVs) without a carrier based on the absence of Wntless (Wls). However, Wls absence does not exclude other transporters or binding partners, such as CRD-containing proteins like Fz, Fz2, ROR, or Otk/Otk2. Performing, or highlighting its need in future work, proteomics of the WgLV may be necessary.
3. Definition of a new vesicle class ("WgLVs")

The introduction of a new term for Rab4/Rab7-positive vesicles is not fully justified. Rab4 and Rab7 compartments are well-established, and double-positive compartments require functional validation before being defined as distinct entities. Colocalisation alone is not sufficient evidence for the classification of a novel organelle or organelle-like structure.

In the discussion, authors suggest that Rab7+ WgLVs could be trafficked to the lysosome for degradation, whereas Rab4+ WgLVs may be more important for transcytosis to the basal membrane. Could the authors confirm this role of Rab7+ vesicles by co-staining with lysosomal markers? And in light of the Rab7+ and Rab4+ compartments having slightly different roles, can they both be labelled under the name WgLV, or do they have distinct purposes?
4. Lack of a clear mechanism for Wg extraction from Wls

The manuscript spends considerable effort explaining how Wg dissociates from Wls and becomes membrane-associated before basolateral release. However, no mechanistic model for this specific step is proposed. The introduction needs be re-phrased to better elucidate the aims and conclusions of the work.
5. Ceramide loss / Schlank KD phenotype interpretation

The Schlank data are intriguing but mechanistically ambiguous. Ceramide depletion could affect:
- Wg trafficking in producing cells,
- Membrane microdomain organization,
- Receptor clustering and signalosome formation in responding cells.

General considerations:
6. Over-reliance on apical imaging

The study relies heavily on apical views. Given that the model depends on polarized trafficking and basolateral release, orthogonal (XZ) sections and basolateral markers should be consistently presented.

Quantification should distinguish:
- Intensity at the source
- True gradient distribution in receiving cells.
7. Novelty and positioning relative to previous work

The tools developed in this study are exceptional; however, they were used for confirmational purposes, rather than generating fresh insights into Wg trafficking. For example, the novel optogenetic tools to inactivate Clathrin are excellent. However, these experiments demonstrate a requirement for endocytosis in Wg release, something which

was previously published and established using the shibirets mutant. Importantly, they do not provide novel insight into Wg-WLS dissociation, and key elements of the model (Rab involvement, glypican function, Schlank effects) have been published previously. The manuscript would benefit from a clear statement of what is genuinely new versus confirmatory.
8. Transferability to other systems

The conclusions are derived exclusively from the wing imaginal disc epithelium. The manuscript should discuss limitations in transferability:
- Many Wg/Wnt-producing tissues are not classical polarised epithelia.
- Different Wnts and tissues use diverse carrier systems.
- Vertebrate systems involve additional extracellular Wnt-binding proteins and transport modes.
9. **Minor comments**

The authors could elaborate further on Figure 9 in the text, which is only briefly discussed. Could the authors suggest why Dlp overexpression cannot rescue the Wnt gradient in the schlank KD, despite rescuing the Wg aggregate formation at the apical membrane?

For Figures 8A & 9, it would be helpful for the reader to have a control image, as these images are lower magnification than the previous figures.

In Figure 4D, could the authors provide an overlay of the GFP-Chc and Cry2oligVHH channels?

In the text, the authors suggest that they assessed the localisation of a panel of endocytic markers with WgLVs. Could the authors expand on the markers that they tested and include some examples in the supplemental data?

There are some instances where "in vitro" is not appropriately italicised. The formatting of this and similar phrases should be double-checked.

2. Significance:

Significance (Required)

General assessment: This study addresses a central question in Wg/Wnt biology by examining how Wg is trafficked across the polarised wing disc epithelium and how endocytosis contributes to its release and signalling range. The work is particularly

valuable for its development of elegant new optogenetic tools to acutely perturb Clathrin function, which could become broadly useful for analysing membrane trafficking in vivo.

Advance: The manuscript proposes an interesting model in which Wg is re-endocytosed apically, separated from Wls, and subsequently redistributed towards the basal side; however, the main conceptual advance is currently limited by unresolved vesicle identity, overinterpretation of Wg as "alone" in these compartments, and a lack of mechanistic clarity on how dissociation from Wls occurs. The study would be strengthened by more rigorous validation of the proposed vesicle classes, clearer positioning of what is genuinely new versus confirmatory, and stronger orthogonal evidence for basolateral trafficking.

Audience: The work will be of interest to researchers in developmental biology, membrane trafficking, and Wnt signalling, particularly those studying epithelial polarity and morphogen dispersal, while the transferable optogenetic tools may also appeal to a wider cell biology community.

3. How much time do you estimate the authors will need to complete the suggested revisions:

Estimated time to Complete Revisions (Required)

(Decision Recommendation)

Between 1 and 3 months

4. *Review Commons* values the work of reviewers and encourages them to get credit for their work. Select 'Yes' below to register your reviewing activity at Web of Science Reviewer Recognition Service (formerly Publons); note that the content of your review will not be visible on Web of Science.

Web of Science Reviewer Recognition

Yes

Review #3

1. Evidence, reproducibility and clarity:

Evidence, reproducibility and clarity (Required)

Summary

This paper proposes new insights into the pathway by which fatty acid-modified Wg is secreted from the basal side in Wg-producing cells of Drosophila.

Specifically, the following results were revealed:

(1) Most of the Wg taken into cells by endocytosis is located in Rab4 and Rab7-positive endosomes, called WgLV.

(2) Inhibition of ceramide synthesis leads to the formation of Wg punctae different from those of WgLV within the cell.

(3) Purified GFP-Wg is incorporated into the outer membrane of liposomes, suggesting that the fatty acid residues of Wg directly penetrate the lipid membrane, and that aggregates are formed in the absence of the detergent.

(4) Inhibition of Dlp, one of the glypicans in Drosophila, leads to the formation of punctae on the basal surface of Wg-producing cells, indicating that Dlp is necessary for the transfer of Wg from WgLV to the basolateral membrane. Furthermore, this inhibition enhances the phenotype caused by of impaired
As a results, the authors propose that ceramide synthesis modulates the orderly transfer of Wingless to the inner endosomal surface thus preventing aggregation and ensuring seamless secretion in the basolateral space.

Major comments

Aside from a few somewhat speculative statements in the abstract, the conclusions in the text for each experiment are convincing. The following points are somewhat speculative and should be revised if possible.

(1) Page1 last line; "perhaps because of a distinct lipid composition there" ---If there is no clear evidence that the ceramide composition of the cell membrane differs from that of the endoplasmic reticulum or Golgi apparatus, this statement is too speculative and should be removed.

(2) The next sentence "Indeed, similar looking puncta are produced upon genetic abrogation of the ceramide synthase Schlank,,," ---While WgLV and intracellular puncta

are distinguished, speculation about other puncta should be made cautiously. While the inference that the puncta observed in the schlank mutant are similar to the aggregates formed in vitro may not be wrong, there is no guarantee that the puncta observed on the apical side due to endocytosis inhibition are similar aggregates. If ceramide synthesis does not change due to endocytosis inhibition, it is difficult to conclude that secreted Wg aggregate simply by inhibiting endocytosis. Rather, it is possible that active (i.e., undenatured) Wg proteins are locally aggregated on the cell membrane, as indicated in the vertebrate (Mii et al 2017)

Minor comments:

- Quantify the amount of Wnt in CM using Fig. 5D WB. Without this data, it is impossible to distinguish whether ceramide synthesis inhibition affects a decrease in Wg secretion or inactivation.
- Quantitative analyses of WgLV: In some experiments, no quantitative analysis directly showing that WgLV is affected has been presented. If such data is presented, it will further support the conclusions. For example, Fig. 5A and Fig. 9.
- Page 4, fourth line from the bottom: "They were also recognizable in imaginal discs,,," If a tag-derived signal is not obtained when using Wg with an impermeable SNAP tag without permialization, the possibilities are either that it is not secreted extracellularly or that it is secreted but remains inside a vesicle. It is unnatural not to mention the former possibility at all. I think it should be mentioned that only the latter possibility exists after a proper explanation.
- Page 6, line 4: "at the apical basal membrane"; Is this a mistake for "at the apical membrane"?

2. Significance:

Significance (Required)

General assessment and Advances

Wnt/Wg is transported to target cells after being modified with fatty acids in the Wnt/Wg-producing cells (Willert et al 2003, Takada et al. 2006). In this transport process, hydrophobic fatty acid residues need to be shielded from the aquatic environment, but how this shield and transport actually occurs is still not fully understood, although various models have been proposed (many reviews including Routledge D, Scholpp S. 2019).
The authors have been working on this problem for a long time using Drosophila wing disc as a model system, and have revealed that Wg, once secreted from the apical side,

undergoes endocytosis and is re-secreted from the basolateral side (Yamazaki et al, 2016). Based on these findings, this paper proposes new insights into the pathway by which fatty acid-modified Wg is secreted from the basal side in Wg-producing cells of Drosophila. This paper newly clarifies the details of the transport process in which Wg, taken into cells by endocytosis, is secreted from the basal side, and is of great significance in understanding the intercellular transport mechanism of Wnt/Wg. In particular, the discovery that Wg is transported within cells by Rab4/Rab7-positive endosomes, which had not been previously identified (Pepperl et al. 2013), is a completely new finding. In addition, it is important that the relationship between ceramide synthesis and Wg transport, which had been shown to be necessary for Wnt signaling but whose detailed mechanism of action had not been clarified, has now been revealed.
On the other hand, the major question raised by the authors in this paper-how fatty acid-modified Wnt is transferred from its complex with the intracellular chaperone Wls while protecting its hydrophobic lipid moiety from the aquatic environment-remains a question that has not yet been fully answered. While it is clear that normal ceramide synthesis is necessary for this transfer, the authors have not presented definitive data on the specific mechanism by which the transfer occurs, such as its incorporation into the lipid bilayer. In that sense, this paper succeeds in elucidating parts of the unknown mechanism involved in intercellular Wnt transfer, but it does not reveal the entire picture.

Audience

This discovery will have a significant impact on developmental biologists and physiologists interested in the intercellular transport of Wnt.

The reviewer's area of expertise is developmental biology of vertebrates, particularly research on the mechanisms and significance of intercellular transport of Wnt ligands.

3. How much time do you estimate the authors will need to complete the suggested revisions:

Estimated time to Complete Revisions (Required)

(Decision Recommendation)

Less than 1 month

4. *Review Commons* values the work of reviewers and encourages them to get credit for their work. Select 'Yes' below to register your reviewing activity at Web of Science

Reviewer Recognition Service (formerly Publons); note that the content of your review will not be visible on Web of Science.

Web of Science Reviewer Recognition

Yes

Revision Plan

Manuscript number: RC- 2026-03434
Corresponding author(s): Jean-Paul Vincent, Ines Alvarez-Rodrigo

[The "revision plan" should delineate the revisions that authors intend to carry out in response to the points raised by the referees. It also provides the authors with the opportunity to explain their view of the paper and of the referee reports.

The document is important for the editors of affiliate journals when they make a first decision on the transferred manuscript. It will also be useful to readers of the reprint and help them to obtain a balanced view of the paper.

If you wish to submit a full revision, please use our "Full Revision" template. ***It is important to use the appropriate template to clearly inform the editors of your intentions.****]*

1. General Statements [optional]

This section is optional. Insert here any general statements you wish to make about the goal of the study or about the reviews.

We are grateful to the reviewers for their helpful comments. Reviewer 1 is positive about the technical strengths of our paper, and both Reviewers 1 and 3 found our conclusions novel, interesting, and important for our field. Reviewer 3 mentioned that our paper "succeeds in elucidating parts of the unknown mechanism involved in intercellular Wnt transfer, but it does not reveal the entire picture". This is a fair assessment. Open questions remain but addressing them will require substantial follow-up work. Reviewer 2 raised questions about the novelty of our findings. However, we feel that this reviewer's summary of our findings needs clarification on two important points:

- Dissociation of Wg from Wls happens prior to re-endocytosis, not after. This is important since it was previously suggested that dissociation occurs in mature endosomes.
- Wls does not accumulate in Rab4+ and/or Rab7+ WgLVs. Since dissociation does not occur in mature endosomes and is not driven by changes in pH, we propose (Fig.10) that Wls and Wg are sorted away from each other soon after endocytosis: Wls enters the recycling pathway, and Wg enters WgLVs.

We thank all three reviewers for highlighting the usefulness of the tools we have developed, in particular the optogenetic tool to inactivate clathrin.

In the sections below we address the reviewers' major and minor points.
Reviewer 1: 0 major points, 5 minor points
Reviewer 2: 8 major points, 5 minor points
Reviewer 3: 2 major points, 4 minor points

We will refer to raised points as for example: R1-MA1 (for major point 1 of reviewer 1) or R1-mi1 (for minor point of reviewer 1). Our response to reviewer 2's major point 1 was subdivided in 3 sections, R2-MA1(a), R2-MA1(b), and R2-MA1(c):

2. Description of the planned revisions

Insert here a point-by-point reply that explains what revisions, additional experimentations and analyses are planned to address the points raised by the referees.

R2-MA1(b) and **R3-mi1**: *Western blot of Wnt3a in conditioned media from L-cells treated with FTY72.*

It is a common experience among Wnt researchers that Wnt3a is difficult to detect in western blots from conditioned media. We will be testing different Wnt3a antibodies and will try the protocol from (Sonavane and Willert, 2022), but we cannot be sure that this will solve the problem.

3. Description of the revisions that have already been incorporated in the transferred manuscript

Please insert a point-by-point reply describing the revisions that were already carried out and included in the transferred manuscript. If no revisions have been carried out yet, please leave this section empty.

R1-mi2: *"Could the authors clarify whether there is less Wg in WgLVs upon schlank knockdown and why the Wg signal is so strong in all the sections in the knockdown region?"*

Upon *schlank* knockdown, some WgLVs still form, albeit in reduced numbers. Numerous structures lacking a lumen also appear (Fig5A''). We suggest that they constitute insoluble aggregates because they disappear upon Dlp overexpression (Fig9). R1 is correct that there is increased Wg signal in the knockdown domain. This is mostly accounted for by the abnormal punctae, which appear throughout the apical-basal axis. We suggest that these aggregates accumulate in Wg-producing cells because they are not readily progressing to the cell surface for release (Fig5E-E''). To further support our assertion that Wg targeting to WgLVs is disrupted upon ceramide synthase knockdown, we include FigS4A-C (lines 231-234 of the Results), which show that accumulated Wg after *schlank* knockdown does not colocalise with Rab7 and Rab4. In addition, we see a marked increase in Wg-positive objects that are not marked by Rab4. We conclude therefore that the formation of WgLVs, which are marked by Rab7 and Rab4, is impaired by *schlank* knockdown~~.~~

R1-mi3: *Quantification of the images in Fig6A to show that Dlp co-localises with some, but not all, WgLVs.*

In Fig6A we show 6 different WgLVs (they all have a visible lumen). It is true that the top 3 are larger than the bottom 3. They were chosen at random from a panel of WgLVs to illustrate that some contain Dlp and others don't, irrespectively of whether they colocalise with Rab4 or not.

Dlp co-localisation was assessed blindly (without prior knowledge of Rab4 immunoreactivity). We have now added the quantification of these data (see Fig6A). R1 is correct that among the 6 examples shown in Fig6A, there seems to be a correlation between the presence of Dlp and WgLV size. We are therefore improving our analysis pipeline to perform a three-way comparison between WgLVs size, Rab4 colocalisation and the presence of Dlp.

[Linked to the previous points] **R3-mi2:** *Quantification of WgLVs, for example in Fig5A and Fig9.*
As indicated in the response to R1-mi2, to address the effect of *schlank* knockdown on WgLVs (Fig5A) we have added quantification in FigS4C albeit not from the same dataset since the presence of YFP-Rab4 was required.
The main aim of Fig9 was to show the effect on abnormal Wg punctae, not to directly look at WgLVs, so the dataset was not imaged with superresolution and we cannot provide a WgLV quantification. Quantification of Wg aggregates rescue by Dlp could not be blinded because the genotype was too obvious from the phenotype.
In addition, linked to R1-mi3, we are now including additional quantification in Fig6A about the presence of Dlp in WgLVs.

R1-mi4: *"Fig.6C - I think I've also missed why in the dlp[KO] experiment, the wt control region (green) has a much higher WgLV density than the three wt controls (gray). If "WgLVs form in the absence of Dlp (dlp[KO]) ... within the wild-type range", shouldn't all conditions look the same?"*
R1 has not missed the explanation; details were lacking. WT1, WT2, WT3 correspond to WgLVs from wild-type (w1118) wing discs obtained in 3 independent experiments. The WgLV density is the same in the *dlp[KO]* compartment as in WT1-3, i.e. "within the wild-type range". WgLV density in the anterior (Dlp-expressing) compartment is unexpectedly higher than in WT1-3. This is possibly due to the *Minute* mutation used to generate the wholly *dlp[KO]* posterior compartment. We have updated the diagram in Fig6B to highlight this, reworded the legends of the graphs in Fig6C to indicate that the anterior compartment is not wild-type, and included a clarification in the figure legend.

R2-MA2: *Overinterpretation of "Wg Alone" in WgLVs - The authors conclude that Wg is present in Wg-lined vesicles (WgLVs) without a carrier based on the absence of Wntless (Wls). However, Wls absence does not exclude other transporters or binding partners, such as CRD-containing proteins like Fz, Fz2, ROR, or Otk/Otk2. Performing, or highlighting its need in future work, proteomics of the WgLV may be necessary.*
We were careful not to use the term "Wg Alone", as we have no evidence that Wg in WgLVs is without a carrier. We can however make the following three points:
- Wg in WgLVs is not bound to Wls, i.e. dissociation from Wls happens prior to Wg reaching WgLVs.
- Dlp is not necessary for Wg to accumulate in WgLVs.
- Incorporation of Wg in liposomes suggest that the Wg lipid can be accommodated by a lipid bilayer.

We have not comprehensively assessed the localization of other lipid shielding proteins at WgLVs, although we found that Fz2, like Dlp, can localise at WgLVs, but not frequently. Otk1/2 are not expressed in wing dics (Linnemannstöns, 2013) while Ror is only expressed in small proneural clusters (Ripp et al., 2018). We cannot rule out the possibility that another protein shields the Wg lipid at WgLVs. Nevertheless, our liposome data suggest that such a protein may not be necessary. We have clarified this position in the text (lines 325-326 of the Results section and 382-387 of the Discussion). Proteomic surveys of intracellular vesicles derived from cultured cells are known to be technically challenging. The same analysis for WgLVs obtained from wing imaginal discs is downright impossible, at least with current technology.

R2-MA4: *No mechanistic model for the step of Wg dissociation from Wls is proposed.*
In our Discussion we suggest that, based on our results and the published structure of Wnt-Wls complexes, “altered lipid composition could lower occupancy of Wls’ central cavity by a specific lipid” causing premature release. We have expanded on this idea further in-text (lines 430-458 of the Discussion) and in the modified Fig10.

[Linked to the previous point] **R3-MA1:** *"Perhaps because of a distinct lipid composition there" – is there evidence that the lipid composition differs between ER, Golgi, plasma membrane?*
Yes, the composition of the cell membrane is different from ER and Golgi with regards to several lipids relevant to this study (van Meer and de Kroon, 2011). This line is no longer in the abstract, which was re-written. The changes made to Fig10 and the Discussion to address R2-MA4 also include evidence from the literature on the different lipid composition of these membranes and its relevance to our model.

R2-MA6: *“Over-reliance on apical imaging” - orthogonal (XZ) sections and basolateral markers should be consistently presented. Quantifications should distinguish intensity at the source and true gradient distribution in receiving cells.*
In most of our figures, three focal planes apical, lateral and basal are displayed. This is not true for Fig1F and Fig3E, which make a point about apical localization. The full complement of focal plane was also not shown in Fig. 4C, 4D, and S4D because of space constraints. In the revised manuscript, we have made space to fit an extra row of images in Fig4C, but space is still an issue for Fig4D and FigS4D, where we show the two most relevant focal planes. We tend to avoid XZ projections because of the reduced axial resolution and increased variability. However, to address R2’s concern, we have added orthogonal projections in Fig3C and Fig5E, where we agree that they will be helpful. We do not see the benefits of quantifying the gradient since the focus of our paper is solely on Wg-producing cells. Indeed, only the signal in source cells is quantified in Fig5E. This was clarified in the legend.

R2-MA7: *Clarify novelty and position relative to previous work regarding (1) requirement for endocytosis for Wg release, (2) insight into Wg-Wls dissociation and (3) key elements of the model (Rab involvement, glypican function, Schlank effects).*

(1) R2 is correct that endocytosis had been previously shown to be important for Wg secretion from Wg-producing cells. We feel that our paper takes significant steps beyond

this conclusion. First, we show that dissociation from Wls can occur in the absence of endocytosis. Moreover, optogenetic inhibition of Clathrin allows us to make the point (lines 403-406; Discussion) that an increase in membrane curvature is not required for Wg release from Wls (since Clathrin is required for the formation of endocytic pits). We have further clarified the difference between the two tools used to inhibit endocytosis in lines 198-199.

(2) Our finding that Wg dissociates from Wls at the plasma membrane, before being targeted to endosomes also argues against the notion that acidification is the main driver of Wg-Wls dissociation. This was mentioned in the Discussion (line 406) and also now highlighted in the results section (lines 168-169).

(3) Double positive Rab4 and Rab7 vesicles have not been reported before in the context of Wg secretion. We show that Schlank has an essential role in Wg-producing cells, not in signal transduction as previously thought. With respect to glypicans, while it is true that the role of Dlp in extracellular gradient formation was known, the formation of basal Wg punctae in *dlp* mutants suggest also a role in Wg export from Wg-producing cells (lines 304-306).

R2-MA8: *Transferability to other systems regarding (1) many Wg/Wnt-producing tissues are not classical polarised epithelia, (2) diversity of Wnt carriers, binders and extracellular transport modes.*

(1) We thank R2 for bringing up the issue of Wnt-production from non-polarized cells. We have added it to our Discussion (lines 459-469).

(2) The diversity of Wnt extracellular transporters is only tangentially relevant to the core message of our paper since we focus on secretion and Wls. The only Wnt that does not require Wls is the unlipidated, arthropod-specific, WntD.

R2-mi1: *Explain the effect of Dlp in Fig9.*

Our results are consistent with previous reports on the effect of wild-type Dlp overexpression on Wg distribution and Wg signaling. We have explained this further in the Results section (lines 339-356) and added extra labels and images to Fig9 to make it easier to follow.

R2-mi3: *In Figure 4D, could the authors provide an overlay of the GFP-Chc and Cry2oligVHH channels?*

Done.

R2-mi4: *In the text, the authors suggest that they assessed the localisation of a panel of endocytic markers with WgLVs. Could the authors expand on the markers that they tested and include some examples in the supplemental data?*

These examples were provided in the initial submission (FigS3A). A reference to FigS3A was added at the beginning of the relevant section (line 150).

R3-MA2: *Amend the sentence from the abstract: "Indeed, similar looking puncta are produced upon genetic abrogation of the ceramide synthase Schlank,,,"*

This was amended in the revised abstract.

R3-mi3: *On the different potential interpretations of the impermeable SNAP tag data regarding WgLV identity*

We realise that the quoted sentence relies on subsequent results with endocytic markers, explaining the reviewer's query. To improve logical flow, we have made two amendments in the Results section:

- Changing the quoted sentence: "suggesting that they are vesicular in nature (intracellular or extracellular) and that Wg molecules are associated directly or indirectly with their limiting membrane (FigS1C)" (lines 134-135).
- And later, after showing the data in Fig2A-C: "We infer therefore that WgLVs are intracellular and that Wg lines their inner membrane, while Rab4 and/or Rab7 coat the outside (FigS3B)" (line 155).

R3-mi4: *Page 6, line 4: "at the apical basal membrane"; Is this a mistake for "at the apical membrane"?*

Yes, this was a mistake that we corrected.

4. Description of analyses that authors prefer not to carry out

Please include a point-by-point response explaining why some of the requested data or additional analyses might not be necessary or cannot be provided within the scope of a revision. This can be due to time or resource limitations or in case of disagreement about the necessity of such additional data given the scope of the study. Please leave empty if not applicable.

R1-mi1: *punctae and not puncta.*

We thank the reviewer for the clarification on the correct spelling of "puncta/ae". We will maintain "punctae" unless the journal's style guide requires otherwise.

R1-mi5: *the Fig.7D images "look like almost wholly black panels with very rare white pixels that are hard to see".*

Unfortunately, the nature of these images makes them tricky to show in other way. The sparsity of Wg signal, particularly in the 1% CHAPS samples, is inevitable. If we zoomed in on rare aggregates, we might give a wrong impression of the frequency of such particles. We are reluctant to use markedly different look-up table for the detergent and non-detergent samples because of difference in background intensity (as indicated in the Figure preparation subsection of Methods). Because the quantification in Fig7E clearly shows the results, we prefer to leave these images as they are.

R2-MA1(a): *vesicle identity remains unclear, the structures could represent stalled carriers or EVs, aberrant endosomal subdomains, membrane blebs with altered composition.*

Staining with endocytic markers show concentric topology (Rab4 and/or Rab7 on the outside, Wg on the inner edge), and the *shibire*^{ts} experiment shows that Wg moves into the WgLVs

within minutes. We think this is enough evidence that WgLVs are endocytic in nature and to rule out the reviewer's suggestions.

R2-MA1(c): *imaging of Wnt3a aggregates*
Detection of Wnt3a aggregates in cells is unfortunately not possible with current tools and reagents. One hurdle is the need for an immunostaining-compatible anti-Wnt3a antibody. It would also require that Wnt3a aggregates be retained at the cells surface during the staining protocol (not an issue with wing discs where this is presumably achieved by the extracellular matrix "sandwiched" between the disc proper and peripodial cells).

R2-MA3: *definition of a new vesicle class ("WgLVs") is not justified, can both Rab4+ and Rab7+ vesicles be labelled WgLVs or do they have distinct purposes.*
We did not mean to suggest that *WgLVs* constitute a new vesicle class. The term Wg-lined vesicle (WgLV) is purely operational (vesicles with Wg enriched at their inner periphery). These vesicles happen to sometimes colocalize with both Rab4 and Rab7. As mentioned in the discussion, Yu et al., 2024 also report partial colocalization of Rab4 and Rab7 in oocyte yolk granules. In our view, the generality and significance of Rab4/Rab7 double positive vesicles constitute a possible avenue for research for colleagues in the endosome field.

R2-MA5: *The Schlank data are intriguing but mechanistically ambiguous. Ceramide depletion could affect Wg trafficking in producing cells, membrane microdomain organization, or receptor clustering and signalosome formation in responding cells.*
We suggested two possible mechanisms whereby ceramide depletion could affect Wg trafficking (the premature release and impaired destination hypotheses). It is true that, with available data, we cannot distinguish these possibilities. In response to the reviewer's specific suggestions, we wish to make the following comments. Altering "membrane microdomain organization" could indeed account for the observed phenotype but does not constitute, in our view, a distinct mechanistic explanation. Perhaps the reviewer refers to the possible effect of ceramide depletion on endocytosis. This is an issue that we already addressed in the Discussion (lines 428-430). Regarding possible effects on receptor clustering and signalosome formation, we re-iterate the fact that Wg protein trafficking is altered upon *schlank* knockdown specifically in Wg-producing cells (shown with advanced genetic tools that go well beyond standards in the field). Whether there are additional effects of *schlank* knockdown on Wg signalling in receiving cells is not a subject of this study. To remove any doubt in the reviewer's mind, we now discuss several ways whereby ceramide depletion could affect Wg trafficking (see also our response to R3-MA1).

R2-mi2: *Control images for Figures8A and 9.*
For Fig8A, we are comparing the effects of removing both Dlp and Schlank (middle: *dlp[KO]* + *schlank* knockdown) to the single depletion (left: *dlp[KO]* and right: *schlank* knockdown). In a separate experiment we have compared *dlp[KO]* heterozygotes (equivalent to wild type control) to *dlp[KO]* homozygotes. Since the results do not add much to our paper, we only make them available to the reviewers (See figure R2-mi2). For Fig9, each anterior compartment is a control for the corresponding posterior compartment, and the left column is a control for the middle and right columns.

R2-mi2: Control images for Fig8A

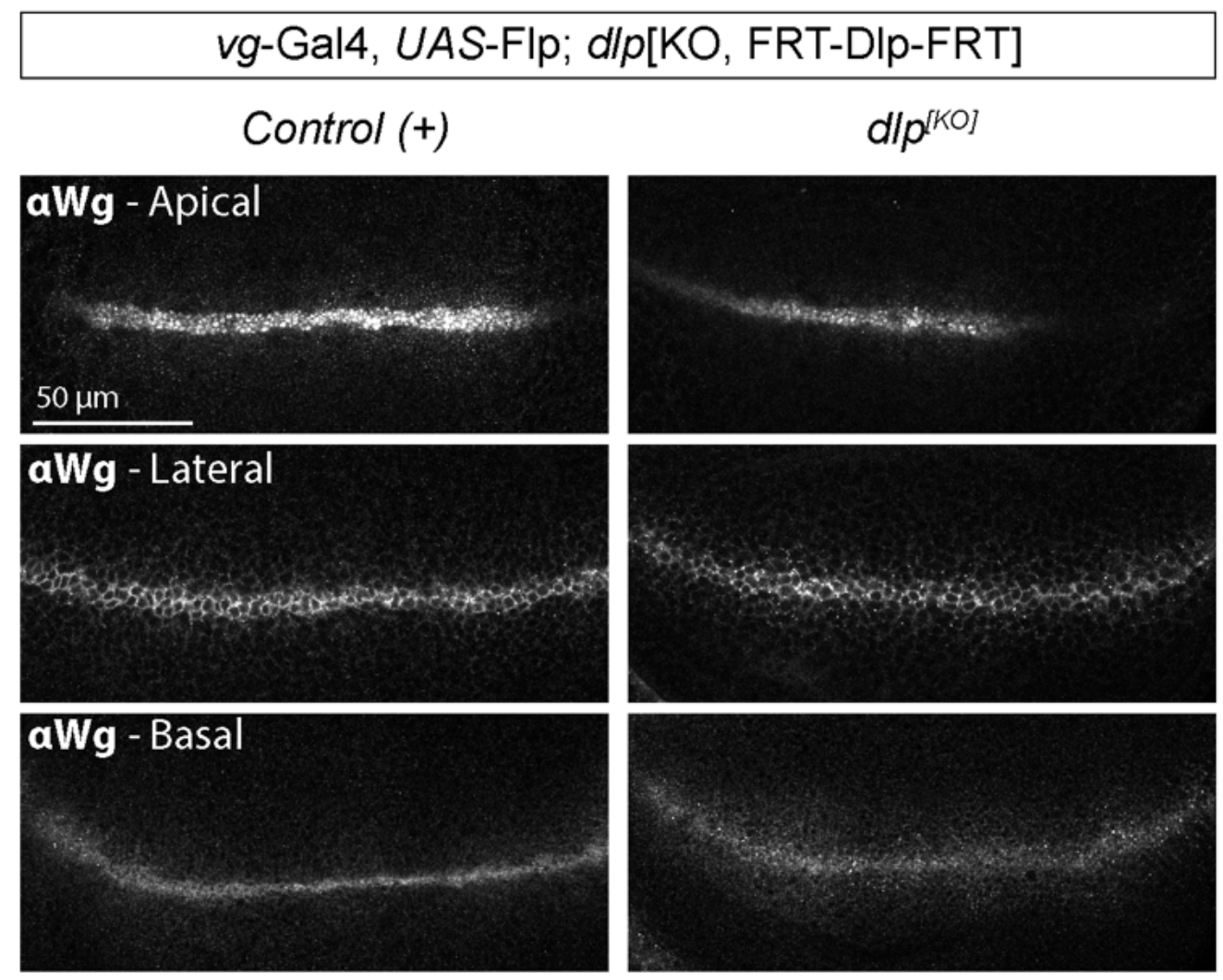

R2-mi5: *There are some instances where "in vitro" is not appropriately italicised.*
As different journals have different style guidelines regarding this, we have changed in vitro to be consistently in regular font and will adapt to the journal style when/if the paper is accepted.

1st Editorial Decision **29th May 2026**

Dear JP,

Thank you submitting a revised version of your manuscript. It was sent to the same three reviewers that originally appraised your work for Review Commons; comments from two of them are attached to the bottom of this email. As you will see, both are satisfied with the changes you made and your plan for a further experiment. There are also some remaining editorial points which need to be addressed. In this regard, would you please:

- indicate the corresponding authors in the main manuscript and provide their email addresses on the title page,
- acknowledge employment in a bitech company in the Disclosure and Competing Interests Statement,
- provide a data availability statement; if no data are provided in external repositories, please state 'This study includes no data deposited in external repositories',
- provide callouts in the manuscript text for panels labeled as A´, A', B', C', D', E', E',
- change the title of the 'Conflict of Interests' statement to the 'Disclosure and Competing Interests Statement',
- acknowledge funding from SFB 645; CNRS, INSERM and ANR in our online submission system,
- provide a completed author checklist,
- upload figures as individual, high-res Figure files with their legends placed below the References in the main manuscript,
- provide an appendix title page containing "Appendix for + ms title" (author and affiliation list not necessary) and a table of contents with the page numbers for the listed items; nomenclature should be Appendix Figure Sx and Appendix Table Sx throughout ms and Appendix PDF; the term "Supplementary" should be removed from the Appendix PDF and callouts in the manuscript,
- provide a Reagents and Tools table,
- provide a synopsis image in .jpg or .png format, exactly 550 pixels wide and 300 pixels high; ensure that the text is legible at the final size and that the synopsis image has appropriate proportions,
- provide suggestions for a short 'blurb' text prefacing and summing up the conceptual aspect of the study in two sentences (max. 250 characters), followed by 3-5 one-sentence 'bullet points' with brief factual statements of key results of the paper; they will form the basis of an editor-written 'Synopsis' accompanying the online version of the article,
- state that images have been reused in Figure 2 A&B and Appendix Fig S3A,
- provide exact p values in the legends of figures 1E; 5D'; 7E; S1-H; S3-C,
- define n and error bars in the legends of figure S1-G,
- define yellow arrowheads in the legend of figure 4D,
- upload Supplementary Table 1-2 as individual files and rename to Figure EV1-EV2 (unless the data should be included in the Reagents & Tools table),
- rename 'Materials and methods' as 'Methods', and
- use the following section order: Title page - Abstract - Introduction - Results - Discussion - Methods - Data Availability - Acknowledgements - Disclosure and Competing Interests Statement - References - Figure Legends - Table(s) - Expanded View Figure Legends.

EMBO Press is an editorially independent publishing platform for the development of EMBO scientific publications.

Best wishes,

William

William Teale, PhD
Editor
The EMBO Journal
w.teale@embojournal.org

Instructions for preparing your revised manuscript:

Read our guidance for manuscript revisions and related editorial policies: https://link.springer.com/journal/44318/submission-guidelines#cms-Revised-submissions

Please also check that the title and abstract of the manuscript are brief, yet explicit, even to non-specialists.

When assembling figures, please refer to our figure preparation guideline in order to ensure proper formatting and readability in print as well as on screen:
https://media.springernature.com/original/springer-cms/rest/v1/content/27825798/data/v1

IMPORTANT: When you send the revision we will require
- a point-by-point response to the referees' comments, with a detailed description of the changes made (as a word file).
- a word file of the manuscript text.
- individual production quality figure files (one file per figure)
- a complete author checklist
- Expanded View files (replacing Supplementary Information)
- a Reagents and Tools Table as part of the Methods section

Please remember: Digital image enhancement is acceptable practice, as long as it accurately represents the original data and conforms to community standards. If a figure has been subjected to significant electronic manipulation, this must be noted in the figure legend or in the 'Methods' section. The editors reserve the right to request original versions of figures and the original images that were used to assemble the figure.

We realize that it is difficult to revise to a specific deadline. In the interest of protecting the conceptual advance provided by the work, we recommend a revision within 3 months (27th Aug 2026). Please discuss the revision progress ahead of this time with the editor if you require more time to complete the revisions. Use the link below to submit your revision:

https://emboj.msubmit.net/cgi-bin/main.plex

Referee #1:

As indicated in my original assessment for Review Commons, this is technically leading-edge work that pushes light microscopy and precise genetic manipulation to their limits. The authors put a great deal of effort into quantifying their data and carrying out statistical analyses (which as far as I can assess, all look appropriate). In addition, to test their hypotheses they also introduce new tools, most notably an optogenetic system to inactivate clathrin, which forms a nice complement to the commonly used shibire[ts] temperature sensitive Dynamin mutation commonly used to block endocytosis. Mechanisms of Wnt function are a complicated and controversial area of research, and this study makes important contributions to our understanding. Particular strengths are that the work is done in an in vivo physiological context, the quality of the imaging and quantitation and the sophisticated genetic manipulations and matching controls that are used.
I only had minor comments on the previous version, and in this revised version these are appropriately addressed. It's also my view that the comments of the other reviewers have been very reasonably addressed, given that I don't think we can expect a complete explanation of Wnt secretion mechanisms from one manuscript. The findings shown are already a significant advance that are worthy of publication.

Referee #2:

The authors' responses to the two major points and four minor points that I raised in my Review Commons review are generally reasonable. Therefore, I see no major issues with proceeding with the revision as proposed.

I would like to add one suggestion regarding their research plan.
R3-mi1: Detecting Wnt in conditioned medium (CM) may be challenging for some researchers; however, successful detection has been reported (For instance, Takada et al, Dev Cell 2006). The authors may consider testing the antibodies used in those studies. An alternative method is to add a tag to Wnt and perform Western blotting using an antibody against the tag.

All editorial and formatting issues were resolved by the authors.

Dear Dr. Vincent,

I am pleased to inform you that your manuscript has been accepted for publication in the EMBO Journal.

Congratulations to you as well as to all involved!

Your manuscript will be processed for publication by EMBO Press. It will be copy edited and you will receive page proofs prior to publication. Please note that you will be contacted by Springer Nature Author Services to complete licensing and payment information.

You may qualify for financial assistance for your publication charges - either via a Springer Nature fully open access agreement or an EMBO initiative. Check your eligibility: https://link.springer.com/journal/44318/how-to-publish-with-us

Should you be planning a Press Release on your article, please notify the production team via this form in order to coordinate publication and release dates: https://forms.cloud.microsoft/e/eH4A8JVP6g

If you have any questions, please do not hesitate to contact the Editorial Office. Thank you for your contribution to The EMBO Journal.

Yours sincerely,

William Teale

William Teale, PhD
Editor
The EMBO Journal
w.teale@embojournal.org

*****
Please note that it is The EMBO Journal policy for the transcript of the editorial process (containing referee reports and your response letters) to be published as an online supplement to each paper. If you should prefer removal of any referee-only figures included in the point-by-point response(s), e.g. because they may still be used for future publication or because they have been reproduced from published work by others, please do let us know immediately via response email.
More information is available here: https://link.springer.com/partners/embo-press/editorial-policies#Peer%20review

** Click here to be directed to your login page: https://emboj.msubmit.net

EMBO Press Author Checklist

Corresponding Author Name: Jean-Paul Vincent, Ines Alvarez-Rodrigo	
Journal Submitted to: The EMBO Journal	
Manuscript Number: EMBOJ-2026-124395-T	

USEFUL LINKS FOR COMPLETING THIS FORM
The EMBO Journal - Author Guidelines
EMBO Reports - Author Guidelines
Molecular Systems Biology - Author Guidelines
EMBO Molecular Medicine - Author Guidelines

Reporting Checklist for Life Science Articles (updated January
This checklist is adapted from Materials Design Analysis Reporting (MDAR) Checklist for Authors. MDAR establishes a minimum set of requirements in transparent reporting in the life sciences (see Statement of Task: 10.31222/osf.io/9sm4x). Please follow the journal's guidelines in preparing your
Please note that a copy of this checklist will be published alongside your article.

Abridged guidelines for figures
1. Data
The data shown in figures should satisfy the following conditions:
- ➞ the data were obtained and processed according to the field's best practice and are presented to reflect the results of the experiments in an accurate and unbiased manner.
- ➞ ideally, figure panels should include only measurements that are directly comparable to each other and obtained with the same assay.
- ➞ plots include clearly labeled error bars for independent experiments and sample sizes. Unless justified, error bars should not be shown for technical
- ➞ if n<5, the individual data points from each experiment should be plotted. Any statistical test employed should be justified.
- ➞ Source Data should be included to report the data underlying figures according to the guidelines set out in the authorship guidelines on Data

2. Captions
Each figure caption should contain the following information, for each panel where they are relevant:
- ➞ a specification of the experimental system investigated (eg cell line, species name).
- ➞ the assay(s) and method(s) used to carry out the reported observations and measurements.
- ➞ an explicit mention of the biological and chemical entity(ies) that are being measured.
- ➞ an explicit mention of the biological and chemical entity(ies) that are altered/varied/perturbed in a controlled manner.
- ➞ the exact sample size (n) for each experimental group/condition, given as a number, not a range;
- ➞ a description of the sample collection allowing the reader to understand whether the samples represent technical or biological replicates (including how many animals, litters, cultures, etc.).
- ➞ a statement of how many times the experiment shown was independently replicated in the laboratory.
- ➞ definitions of statistical methods and measures:
  - common tests, such as t-test (please specify whether paired vs. unpaired), simple χ2 tests, Wilcoxon and Mann-Whitney tests, can be unambiguously identified by name only, but more complex techniques should be described in the methods section;
  - are tests one-sided or two-sided?
  - are there adjustments for multiple comparisons?
  - exact statistical test results, e.g., P values = x but not P values < x;
  - definition of 'center values' as median or average;
  - definition of error bars as s.d. or s.e.m.

Please complete ALL of the questions below.
Select "Not Applicable" only when the requested information is not relevant for your study.

Materials

Newly Created Materials	Information included in the manuscript?	In which section is the information available? (Reagents and Tools Table, Materials and Methods, Figures, Data Availability Section)
New materials and reagents need to be available; do any restrictions apply?	Yes	No restrictions. Data availability section

Antibodies	Information included in the manuscript?	In which section is the information available? (Reagents and Tools Table, Materials and Methods, Figures, Data Availability Section)
For **antibodies** provide the following information:<br>- Commercial antibodies: RRID (if possible) or supplier name, catalogue number and or/clone number<br>- Non-commercial: RRID or citation	Yes	Reagents and Tools Table, Method section

DNA and RNA sequences	Information included in the manuscript?	In which section is the information available? (Reagents and Tools Table, Materials and Methods, Figures, Data Availability Section)
**Short novel DNA or RNA including primers, probes**: provide the sequences.	Yes	Reagents and Tools Table

Cell materials	Information included in the manuscript?	In which section is the information available? (Reagents and Tools Table, Materials and Methods, Figures, Data Availability Section)
**Cell lines:** Provide species information, strain. Provide accession number in repository **OR** supplier name, catalog number, clone number, and/**OR** RRID.	Yes	Reagents and Tools Table, Method section
**Primary cultures:** Provide species, strain, sex of origin, genetic modification status.	Not Applicable	
Report if the cell lines were recently **authenticated** (e.g., by STR profiling) and tested for mycoplasma contamination.	Yes	Methods section

Experimental animals	Information included in the manuscript?	In which section is the information available? (Reagents and Tools Table, Materials and Methods, Figures, Data Availability Section)
**Laboratory animals or Model organisms:** Provide species, strain, sex, age, genetic modification status. Provide accession number in repository **OR** supplier name, catalog number, clone number, **OR** RRID.	Yes	Reagents and Tools Table, Method section
**Animal observed in or captured from the field:** Provide species, sex, and age where possible.	Not Applicable	
Please detail **housing and husbandry conditions**.	Yes	Methods section

Plants and microbes	Information included in the manuscript?	In which section is the information available? (Reagents and Tools Table, Materials and Methods, Figures, Data Availability Section)
**Plants:** provide species and strain, ecotype and cultivar where relevant, unique accession number if available, and source (including location for collected wild specimens).	Not Applicable	
**Microbes:** provide species and strain, unique accession number if available, and source.	Not Applicable	

Human research participants	Information included in the manuscript?	In which section is the information available? (Reagents and Tools Table, Materials and Methods, Figures, Data Availability Section)
If collected and within the bounds of privacy constraints report on age, sex and gender or ethnicity for all study participants.	Not Applicable	

Core facilities	Information included in the manuscript?	In which section is the information available? (Reagents and Tools Table, Materials and Methods, Figures, Data Availability Section)
If your work benefited from core facilities, was their service mentioned in the acknowledgments section?	Yes	Acknowledgements

Design

Study protocol	Information included in the manuscript?	In which section is the information available? (Reagents and Tools Table, Materials and Methods, Figures, Data Availability Section)
If study protocol has been **pre-registered, provide DOI in the manuscript**. For clinical trials, provide the trial registration number **OR** cite DOI.	Not Applicable	
Report the **clinical trial registration number** (at ClinicalTrials.gov or equivalent), where applicable.	Not Applicable	

Laboratory protocol	Information included in the manuscript?	In which section is the information available? (Reagents and Tools Table, Materials and Methods, Figures, Data Availability Section)
Provide DOI OR other citation details if **external detailed step-by-step protocols** are available.	Not Applicable	

Experimental study design and statistics	Information included in the manuscript?	In which section is the information available? (Reagents and Tools Table, Materials and Methods, Figures, Data Availability Section)
Include a statement about **sample size** estimate even if no statistical methods were used.	Yes	Methods section
Were any steps taken to minimize the effects of subjective bias when allocating animals/samples to treatment (e.g. **randomization procedure**)? If yes, have they been described?	Not Applicable	
Include a statement about **blinding** even if no blinding was done.	Yes	Methods section
Describe **inclusion/exclusion criteria** if samples or animals were excluded from the analysis. Were the criteria pre-established?<br><br>If sample or data points were omitted from analysis, report if this was due to attrition or intentional exclusion and provide justification.	Yes	Methods section
For every figure, are **statistical tests** justified as appropriate? Do the data meet the assumptions of the tests (e.g., normal distribution)? Describe any methods used to assess it. Is there an estimate of variation within each group of data? Is the variance similar between the groups that are being statistically compared?	Yes	Methods section, Figure legends

Sample definition and in-laboratory replication	Information included in the manuscript?	In which section is the information available? (Reagents and Tools Table, Materials and Methods, Figures, Data Availability Section)
In the figure legends: state number of times the experiment was **replicated** in laboratory.	Yes	Figure legends
In the figure legends: define whether data describe **technical or biological replicates**.	Yes	Figure legends

Ethics

Ethics	Information included in the manuscript?	In which section is the information available? (Reagents and Tools Table, Materials and Methods, Figures, Data Availability Section)
Studies involving **human participants**: State details of **authority granting ethics approval** (IRB or equivalent committee(s), provide reference number for approval.	Not Applicable	
Studies involving **human participants**: Include a statement confirming that **informed consent** was obtained from all subjects and that the experiments conformed to the principles set out in the WMA Declaration of Helsinki and the Department of Health and Human Services Belmont Report.	Not Applicable	
Studies involving **human participants:** For publication of **patient photos**, include a statement confirming that consent to publish was obtained.	Not Applicable	
Studies involving experimental **animals**: State details of **authority granting ethics approval** (IRB or equivalent committee(s), provide reference number for approval. Include a statement of compliance with ethical regulations.	Not Applicable	
Studies involving **specimen and field samples:** State if relevant **permits** obtained, provide details of authority approving study; if none were required, explain why.	Not Applicable	

Dual Use Research of Concern (DURC)	Information included in the manuscript?	In which section is the information available? (Reagents and Tools Table, Materials and Methods, Figures, Data Availability Section)
Could your study fall under dual use research restrictions? Please check biosecurity documents and list of **select agents and toxins** (CDC): https://www.selectagents.gov/sat/list.htm	Not Applicable	
If you used a select agent, is the security level of the lab appropriate and reported in the manuscript?	Not Applicable	
If a study is subject to dual use research of concern regulations, is the name of the **authority granting approval and reference number** for the regulatory approval provided in the manuscript?	Not Applicable	

Reporting

The MDAR framework recommends adoption of discipline-specific guidelines, established and endorsed through community initiatives. Journals have their own policy about requiring specific guidelines and recommendations to complement MDAR.

Adherence to community standards	Information included in the manuscript?	In which section is the information available? (Reagents and Tools Table, Materials and Methods, Figures, Data Availability Section)
State if relevant guidelines or checklists (e.g., **ICMJE, MIBBI, ARRIVE, PRISMA**) have been followed or provided.	Not Applicable	
For **tumor marker prognostic studies,** we recommend that you follow the **REMARK** reporting guidelines (see link list at top right). See author guidelines, under 'Reporting Guidelines'. Please confirm you have followed these guidelines.	Not Applicable	
For **phase II and III randomized controlled trials**, please refer to the **CONSORT** flow diagram (see link list at top right) and submit the CONSORT checklist (see link list at top right) with your submission. See author guidelines, under 'Reporting Guidelines'. Please confirm you have submitted this list.	Not Applicable	

Data Availability

Data availability	Information included in the manuscript?	In which section is the information available? (Reagents and Tools Table, Materials and Methods, Figures, Data Availability Section)
Have **primary datasets** been deposited according to the journal's guidelines (see 'Data Deposition' section) and the respective accession numbers provided in the Data Availability Section?	Yes	Data availability section
Were **human clinical and genomic datasets** deposited in a public access-controlled repository in accordance to ethical obligations to the patients and to the applicable consent agreement?	Not Applicable	
Are **computational models** that are central and integral to a study available without restrictions in a machine-readable form? Were the relevant accession numbers or links provided?	Not Applicable	
If publicly available data were reused, provide the respective **data citations in the reference list**.	Yes	Figure legend, reference list
