# Supplementary material for: Membrane lipid composition and rapid endocytic retrieval modulate Wingless secretion
Source: EMBO J. 2026 Aug 3;45(17):6139–75. doi: 10.1038/s44318-026-00881-2 (PMC13534635; doi:10.1038/s44318-026-00881-2)

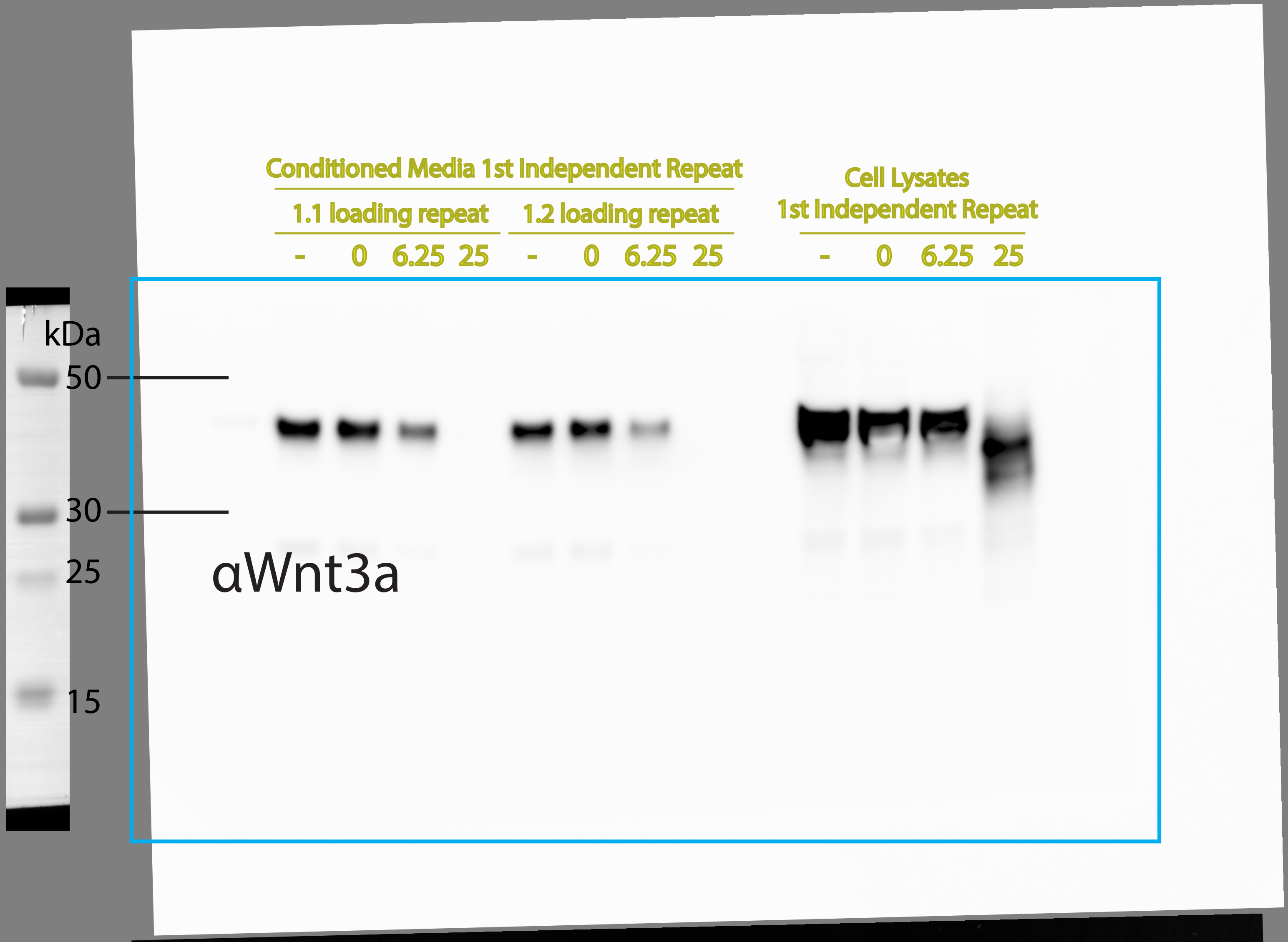

Conditioned Media 1st Independent Repeat
1.1 loading repeat
1.2 loading repeat
- 0 6.25 25
- 0 6.25 25
Cell Lysates
1st Independent Repeat
- 0 6.25 25
kDa
50
30
25
15
αWnt3a

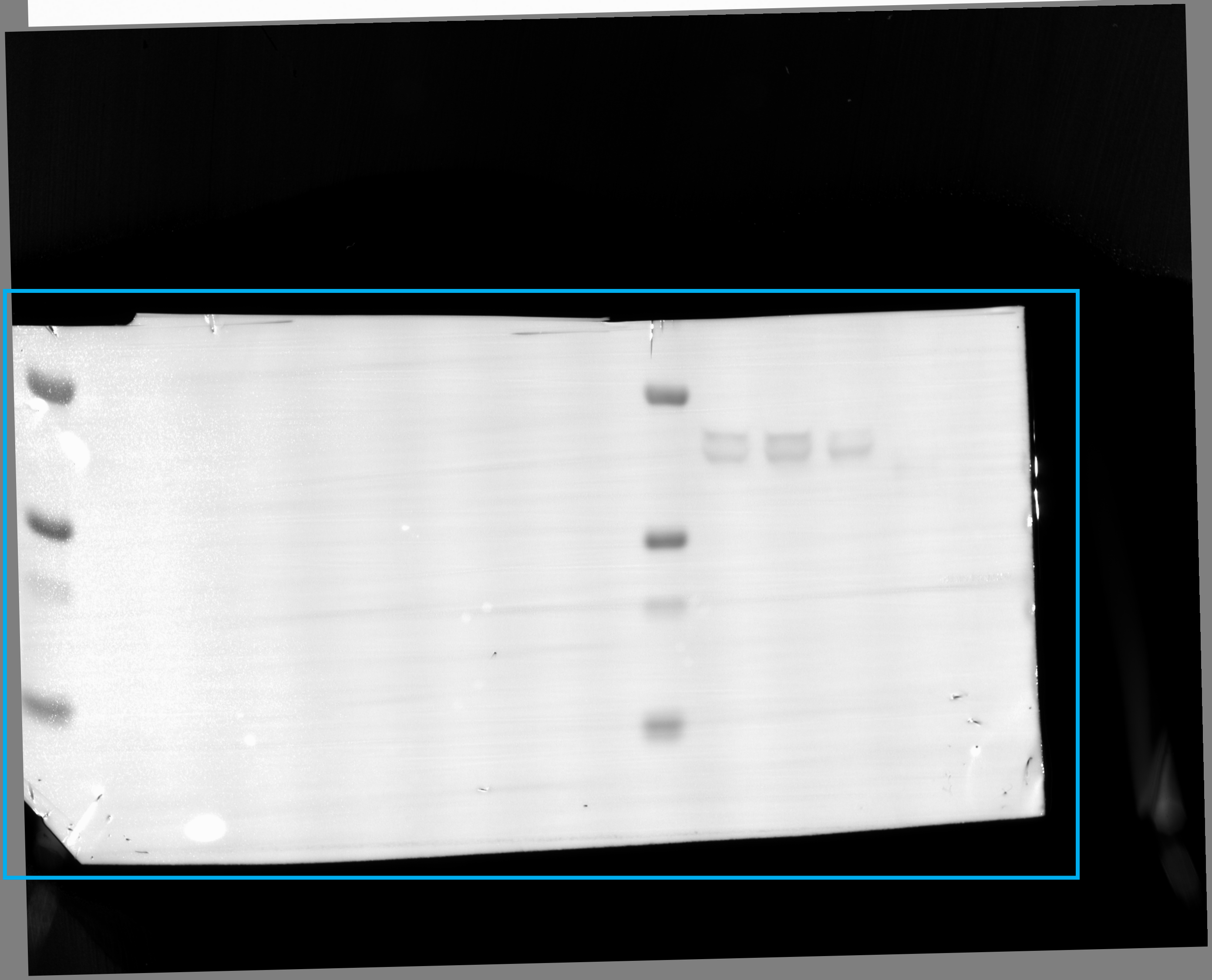

Supplement: Supplementary file 10 [file 44318_2026_881_MOESM7_ESM.zip › Figure 6/6C/260603_repeat_wnt3a cm blot.pdf]
