# Supplementary material for: Membrane lipid composition and rapid endocytic retrieval modulate Wingless secretion
Source: EMBO J. 2026 Aug 3;45(17):6139–75. doi: 10.1038/s44318-026-00881-2 (PMC13534635; doi:10.1038/s44318-026-00881-2)
Supplement: Supplementary file 10 [file 44318_2026_881_MOESM7_ESM.zip › Figure 6/6C/260603_ponceau lysate blot_repeats 2 and 3.pdf]

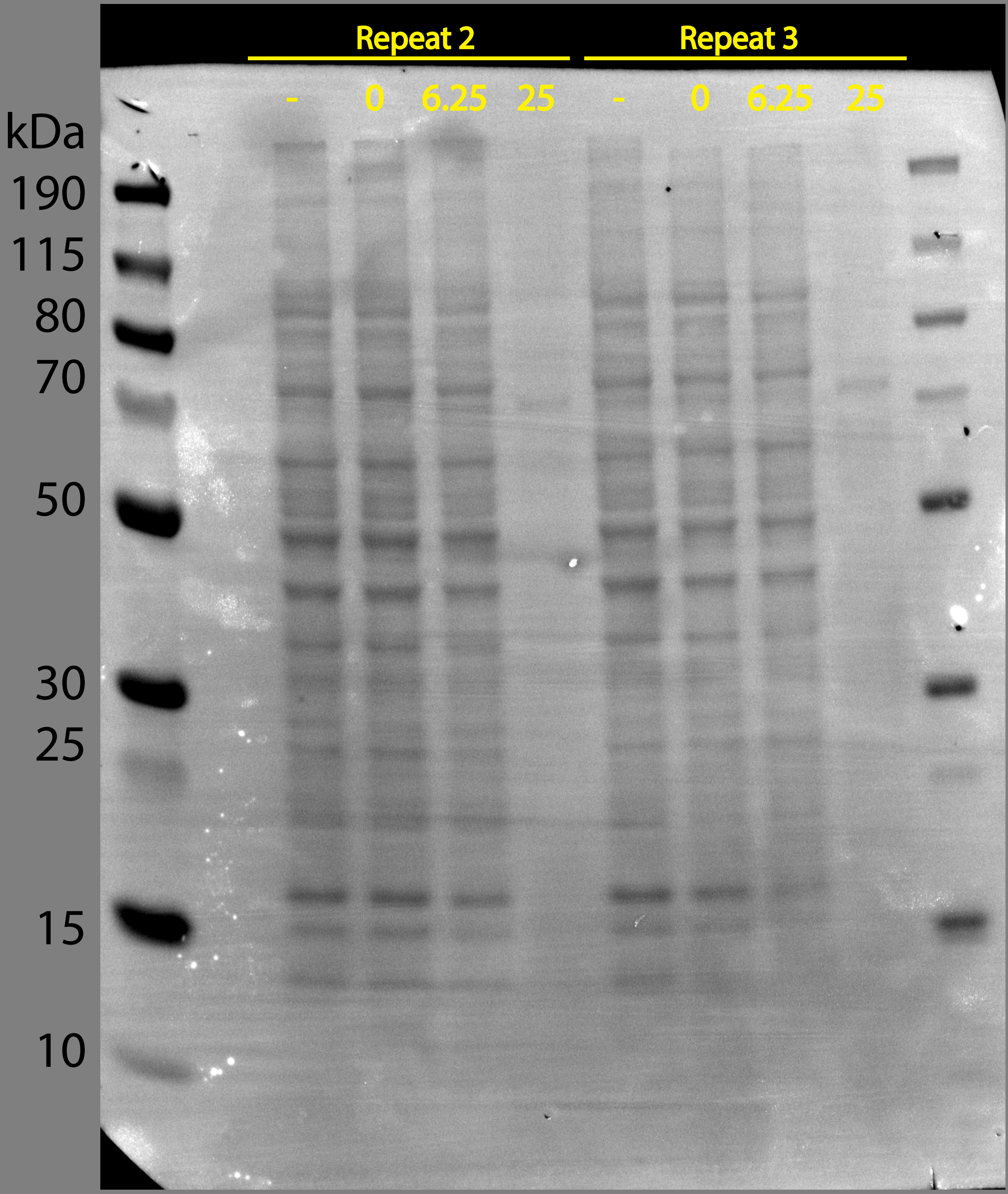

Ponceau - cell lysate blot prior to cutting the membrane for Wnt3a and Calnexin staining
