# Supplementary material for: Membrane lipid composition and rapid endocytic retrieval modulate Wingless secretion
Source: EMBO J. 2026 Aug 3;45(17):6139–75. doi: 10.1038/s44318-026-00881-2 (PMC13534635; doi:10.1038/s44318-026-00881-2)
Supplement: Supplementary file 11 [file 44318_2026_881_MOESM8_ESM.zip › Figure 7/7A/230128_DlpWgLVsQuantifc_IndividualWgLVs.pdf]

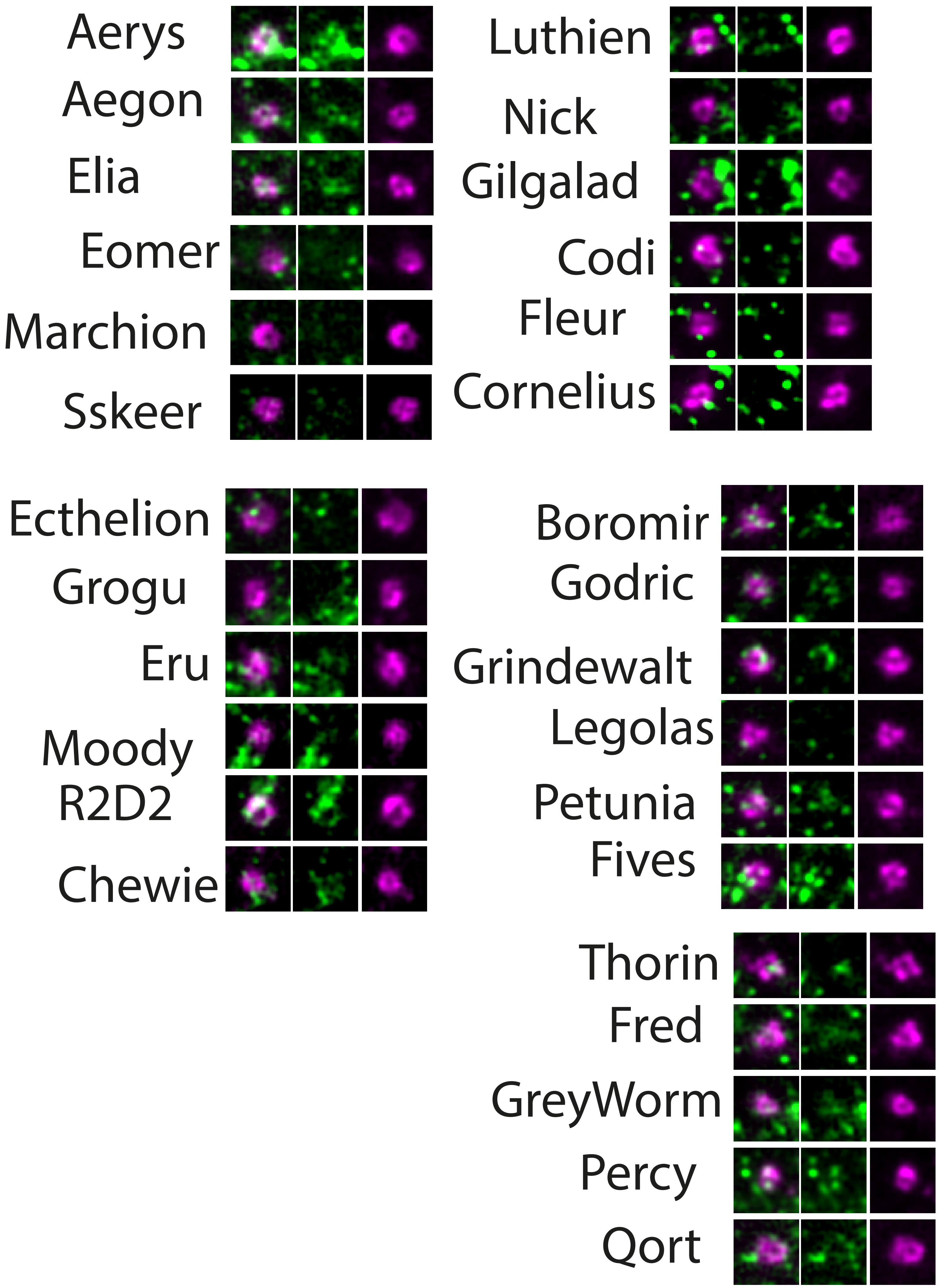
Aerys
Aegon
Elia
Eomer
Marchion
Sskeer
Luthien
Nick
Gilgalad
Codi
Fleur
Cornelius
Ecthelion
Grogu
Eru
Moody
R2D2
Chewie
Boromir
Godric
Grindewalt
Legolas
Petunia
Fives
Thorin
Fred
GreyWorm
Percy
Qort
