# Supplementary material for: Membrane lipid composition and rapid endocytic retrieval modulate Wingless secretion
Source: EMBO J. 2026 Aug 3;45(17):6139–75. doi: 10.1038/s44318-026-00881-2 (PMC13534635; doi:10.1038/s44318-026-00881-2)
Supplement: Supplementary file 12 [file 44318_2026_881_MOESM9_ESM.zip › Figure 8/8A/250903_BlotForFigure_liposomes.pdf]

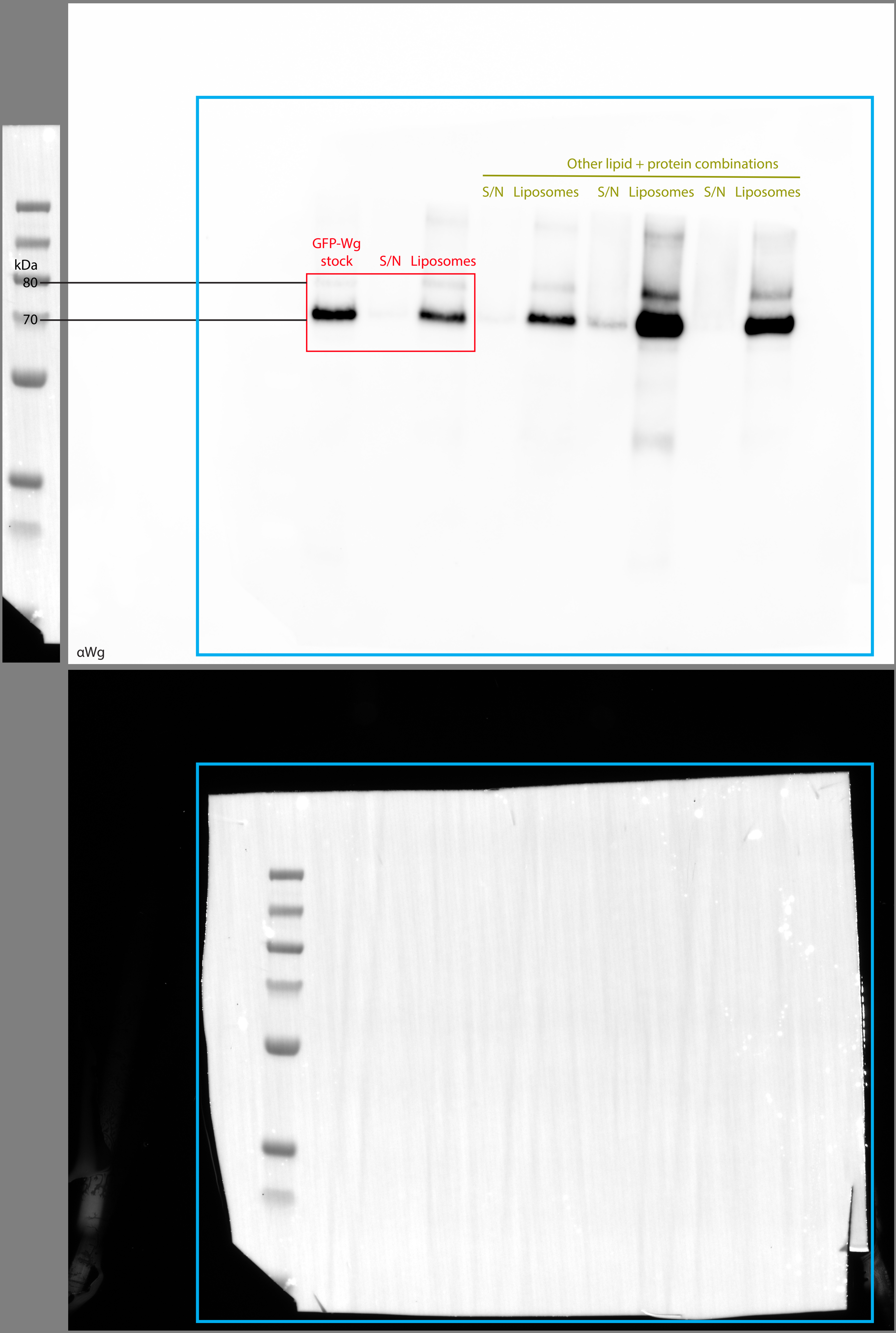

Other lipid + protein combinations
S/N Liposomes S/N Liposomes S/N Liposomes
GFP-Wg stock
S/N Liposomes
kDa
80
70
αWg
